# Ribosome quality control is a central protection mechanism for yeast exposed to deoxynivalenol and trichothecin

**DOI:** 10.1186/s12864-016-2718-y

**Published:** 2016-06-01

**Authors:** Karl G. Kugler, Zeljkica Jandric, Reinhard Beyer, Eva Klopf, Walter Glaser, Marc Lemmens, Mehrdad Shams, Klaus Mayer, Gerhard Adam, Christoph Schüller

**Affiliations:** Department of Applied Genetics and Cell Biology, UFT-Campus Tulln-Konrad, University of Natural Resources and Life Sciences, Vienna (BOKU), Konrad Lorenz Strasse 24, 3430 Tulln an der Donau, Austria; Plant Genome and Systems Biology, Helmholtz Zentrum München, Ingolstädter Landstraße 1, 85764 Neuherberg, Germany; University of Natural Resources and Life Sciences, Vienna (BOKU), Institute for Biotechnology in Plant Production, Konrad-Lorenz-Straße 20, 3430 Tulln an der Donau, Austria

**Keywords:** Mycotoxins, Fusarium, Trichothecenes, Synthetic genetic array, Ribosome, RSC complex, Translation quality control, Deoxynivalenol, Yeast

## Abstract

**Background:**

The trichothecene mycotoxins deoxynivalenol (DON) and trichothecin (TTC) are inhibitors of eukaryotic protein synthesis. Their effect on cellular homeostasis is poorly understood. We report a systematic functional investigation of the effect of DON and TTC on the yeast *Saccharomyces cerevisiae* using genetic array, network and microarray analysis. To focus the genetic analysis on intracellular consequences of toxin action we eliminated the *PDR5* gene coding for a potent pleiotropic drug efflux protein potentially confounding results. We therefore used a knockout library with a *pdr5*Δ strain background.

**Results:**

DON or TTC treatment creates a fitness bottleneck connected to ribosome efficiency. Genes isolated by systematic genetic array analysis as contributing to toxin resistance function in ribosome quality control, translation fidelity, and in transcription. Mutants in the E3 ligase Hel2, involved in ribosome quality control, and several members of the Rpd3 histone deacetylase complex were highly sensitive to DON. DON and TTC have similar genetic profiles despite their different toxic potency. Network analysis shows a coherent and tight network of genetic interactions among the DON and TTC resistance conferring gene products. The networks exhibited topological properties commonly associated with efficient processing of information. Many sensitive mutants have a "slow growth" gene expression signature. DON-exposed yeast cells increase transcripts of ribosomal protein and histone genes indicating an internal signal for growth enhancement.

**Conclusions:**

The combination of gene expression profiling and analysis of mutants reveals cellular pathways which become bottlenecks under DON and TTC stress. These are generally directly or indirectly connected to ribosome biosynthesis such as the general secretory pathway, cytoskeleton, cell cycle delay, ribosome synthesis and translation quality control. Gene expression profiling points to an increased demand of ribosomal components and does not reveal activation of stress pathways. Our analysis highlights ribosome quality control and a contribution of a histone deacetylase complex as main sources of resistance against DON and TTC.

**Electronic supplementary material:**

The online version of this article (doi:10.1186/s12864-016-2718-y) contains supplementary material, which is available to authorized users.

## Background

Trichothecene mycotoxins are toxic sesquiterpenoid secondary metabolites produced by mostly plant pathogenic fungi of the genera *Trichothecium*, *Myrothecium*, *Trichoderma*, and *Fusarium* [1]. Deoxynivalenol (DON), accumulates in infected small grain cereals and is a virulence factor of *F. graminearum* in the course of Fusarium head blight (FHB) disease, potentially causing health problems to humans and animals [2]. Trichothecin (TTC) is produced by the grape dry rot pathogen *Trichothecium roseum* and can be found in red wine [3]. TTC may have mainly antagonistic activity against competing fungi and is more toxic to yeast, presumably due to higher membrane permeability caused by a hydrophobic side chain (isocrotonyl-ester). The primary mode of action of both DON and TTC is inhibition of eukaryotic protein synthesis [4, 5]. Depending on the organism, cell type, exposure and trichothecene derivative exposure can cause a variety of phenotypes. In wheat, DON is a virulence factor for *F. graminearum* required for fungal spread [6] and is produced in the early stages during the host-pathogen interaction [7]. In animals, trichothecenes cause pleiotropic systemic effects ranging from feed refusal to immune suppression [8]. In mammalian cells, DON induces activation of double-stranded RNA-associated protein kinase (PKR), recruits and induces Map kinases (p38, JNK) which in turn activate their downstream targets, and promotes degradation of 28S rRNA [9]. Prolonged DON exposure triggers apoptosis and rRNA cleavage [2]. In plants, trichothecenes modulate abiotic stress signalling and lead to the induction of oxidative stress and cell death [10].

The plant-fungal interaction is complicated with trichothecenes apparently playing several roles in a biological arms race. Genetic analysis of Fusarium-host interaction and trichothecene effects is up to now most thoroughly developed for wheat, barley and maize. In wheat over 100 quantitative trait loci (QTLs) have been described to contribute to resistance [11]. Qfhs.ndsu-3BS, is strongly contributing to *Fusarium* spreading and toxin resistance [12–14] and contains the (yet unidentified) *Fhb1* resistance gene(s). It is likely that inhibition of protein synthesis by DON is not the only factor interfering with plant cellular physiology. DON is required for efficient infection of wheat. *F. graminearum tri5* mutants which are unable to produce DON are prevented from spreading to the next spikelet, however are infectious. Metabolomic studies suggested that trichothecenes and especially DON are not only inhibiting protein biosynthesis but also directly repress plant resistance mechanisms [15, 16]. Another layer of complexity of the fungus-toxin-host system is introduced by the multiple ways of toxin biotransformation. In wheat, glucosylation and glutathione conjugation are major routes of DON biotransformation [17, 18]. The overall role of toxin production for the fungal life cycle is not clear. Plant pathogenic fungi like Fusarium are saprophytes during large parts of the year and trichothecenes might protect infected grains against feeders, thus perhaps improving survival of *F. graminearum* spores and grains. Trichothecene metabolites may also provide protection against fungivores such as mites and insects [19]. Furthermore, they could play a role in the competition with other microbes co-occurring in soil and plant debris or provide protection against mycoparasitic fungi. Nevertheless, the effect of trichothecenes on cells and animals usually not involved in the plant versus fungal warfare is of economic significance. In experimental animal models, acute DON poisoning causes emesis, whereas chronic low-dose exposure elicits anorexia, growth retardation, immunotoxicity as well as impaired reproduction and development resulting from maternal intoxication. Pathophysiologic effects associated with DON include altered neuroendocrine signalling, proinflammatory gene induction, disruption of the growth hormone axis, and altered gut integrity [10].

Despite investigative efforts, the systemic effect of trichothecene toxicity is not well-understood and there is a gap in our knowledge about general mechanisms that can protect cells against trichothecene toxins. Here we investigate the impact of DON and TTC on the simple eukaryotic model organism *Saccharomyces cerevisiae*. Yeast genetics pointed early to the molecular targets of trichothecene toxicity and showed inhibition of eukaryotic translation by targeting the yeast ribosomal protein L3 [20, 21]. The yeast deletion collection is a perfect tool to study effects of drugs in a systematic genetics analysis [22–25] and has even been used for a yeast genetics experiment in space [26]. Two genetic survey studies have been reported identifying yeast mutants resistant and hypersensitive to TTC [27, 28]. These studies both suggested that TTC sensitivity is connected to mitochondrial dysfunction and the resulting intracellular oxidative stress. To gain insights into the DON defence response of a simple eukaryotic cell we screened the yeast deletion collection for sensitive strains and performed gene expression profiling. The DON sensitivity of yeast was increased in our screen by elimination of the drug efflux ABC transporter Pdr5. Interestingly, *PDR5* has not been listed as gene involved in TTC resistance in a previous study [28]. Pdr5 contributes highly to yeast DON and TTC resistance and is controlled by the status of the mitochondria [29]. Thus elimination of this pathway should reveal intracellular hotspots for general trichothecene resistance. Yeast genes isolated here in a background lacking Pdr5 point to cytoplasmic efficiency and rRNA synthesis as rate limiting for fitness in the presence of DON and TTC.

## Results and discussion

**Fig. 1:**
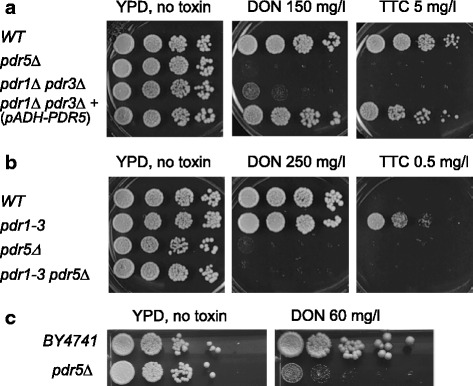
Pdr5 confers TTC and DON resistance. Logarithmic phase cultures in YPD medium were diluted to OD600 0.1. Then 5 μl of this and three serial 1:6 dilutions were spotted on YPD plates containing the indicated amount of toxin. **a** The isogenic set of strains is derived from strain FY1679-28C. **b** The congenic set of strains is derived from YALA-B1 and YALA-G4. See material and methods for strain construction. **c** Pdr5 mediates DON resistance in the mutant strains collection background. BY4741 and BY4741*pdr5*Δ were spotted in serial dilution on DON containing YPD medium

To define the impact of DON on cellular processes we screened the yeast deletion library for mutants exhibiting sensitivity to DON and TTC. A previous study analysed yeast deletion mutants treated with TTC [28]. Such a screen has not been reported for DON due to its low toxicity in yeast. To focus on specific intracellular mechanisms conferring resistance we tried to reduce drug efflux. Our initial observations suggested a major role of the ABC-type drug efflux pump Pdr5 and that this protein is necessary and sufficient to confer resistance to the trichothecenes DON and TTC. In yeast, *PDR5* and numerous ABC transporters and pleiotropic drug resistance genes are regulated by the transcription factors Pdr1 and Pdr3 [30]. Deletion of both genes (*pdr1 pdr3*) leads to higher TTC and DON sensitivity and deletion of *PDR5* causes an even stronger toxin sensitivity phenotype (Fig. 1a). Trichothecene resistance can be restored in a *pdr1 pdr3* double mutant by constitutive high *ADH1* promoter driven expression of *PDR5* alone (Fig. 1a). A gain of function allele of Pdr1 (*PDR1-3)* leads to overexpression of *PDR5* and other drug resistance genes [31] and causes increased resistance to high concentrations of TTC. Deleting *PDR5* in the dominant *PDR1-3* background leads to toxin sensitivity, indicating that other genes upregulated by *PDR1-3* do not play a major role (Fig. 1b). As expected, a deletion of *PDR5* did also confer higher sensitivity against DON in the strain background of the knock out collection (Fig. 1c). Elimination of the *PDR5* gene from the collection was an important step to lower the required DON concentration. A further advantage of the absence of *PDR5* from the knockout collection is that we avoid those mutants which are DON/TTC sensitive due to changed *PDR5* expression. Expression of *PDR5* is not constitutive [32] and subject to retrograde signaling [29]. A conserved mechanism is increasing the expression of the transcription factor Pdr3 in yeast leading to enhanced Pdr5 expression. Similarly, in the related yeast *Candida glabrata* CgPdr1 induces the Pdr5 homolog Cdr1 [33]. Importantly, DON exposure increases *PDR1* and *PDR3* expression [34]. Thus, one difference of our study to previous ones is the insensitivity of our *PDR5* deleted mutant collection to variations of Pdr5 levels.

The library of double mutants was generated using the SGA protocol [35]. The query strain carrying the *PDR5* gene knockout was mated with the strains of the yeast deletion collection. The diploid strains were selected and then sporulated. The desired haploid double mutant segregants were obtained by counter selection against the diploid and other meiotic products [35]. The initial screen for sensitive double mutants of this library was performed with 70 mg/l DON and 0.3 mg/l TTC on solid rich medium and followed by a subsequent rescreening. We identified a total of 248 strains of which 92 were DON sensitive and 197 TTC sensitive (Additional file 1: Table S1 and Additional file 2: Data S1). The 35 deletion strains most sensitive to both DON and TTC are listed in Table 1 (Additional file 3: Data S2). A previous study [28] identified 121 strains sensitive to TTC with 23 overlapping strains reported here (Additional file 4: Data S3). The overlap of identified strains is significant (p ~ 5 · 10^−10^). Many of the mutants found in both studies have also been isolated in many different screens [22, 36]. In order to focus on genes involved specifically in toxin response we marked the notoriously isolated multidrug sensitive mutants in our analysis (e.g. Table 1; MDR). These belong to the CCR4-NOT complex (*CCR4, POP2, VMS1*), cytoskeleton and endocytosis (*RVS161, RVS167, SAC6, VRP1, SLA1*), vacuole (*VMA8, VMA11, VMA13, VMA22*), the cell wall integrity pathway (*SLT2, BCK1*), *URE2* encoding a prion, the sterol desaturase encoded by *ERG3* affecting membrane permeability, the chromatin remodelling complex subunit Snf2, as well as a number of other genes (*LSM1, BUB1, DBF2, MCH5*).

**Table 1:** Selected deletion strains sensitive to both TTC and DON

I	YORF	NAME	DON	Tcin	Term	Function	MDR
5	YMR078C	CTF18	3	3	DNA damage	Subunit of a complex with Ctf8p	
6	YOR299W	BUD7	3	4	Golgi	Member of the ChAPs family	
7	YEL036C	ANP1	3	2	Golgi	Subunit of the alpha-1,6 mannosyltransferase complex	2
8	YPR051W	MAK3	3	2	Golgi	Catalytic subunit N-terminal acetyltransferase	
9	YOR147W	MDM32	4	4	Mitochondria	Mitochondrial inner membrane protein	
10	YOR178C	GAC1	4	4	PP1	Regulatory subunit for Glc7p type-1 protein Pase (PP1)	
11	YCL045C	EMC1	2	4	Folding	Member of conserved ER membrane complex	
12	YIL084C	SDS3	3	4	Rpd3 HDAC	Component of the Rpd3L histone deacetylase complex	
13	YOL004W	SIN3	3	4	Rpd3 HDAC	Component of both the Rpd3S and Rpd3L HDAC	5
14	YPL139C	UME1	3	3	Rpd3 HDAC	Component of both the Rpd3S and Rpd3L HDAC	
16	YOR043W	WHI2	3	3	Stress	Required for activation of the general stress response	
17	YGL025C	PGD1	3	3	Transcription	Subunit of the RNA polymerase II mediator complex	1
18	YOR039W	CKB2	3	2	Transcription	Beta' regulatory subunit of casein kinase 2 (CK2)	
19	YEL007W	MIT1	3	2	Transcription	Transcriptional regulator of pseudohyphal growth	
20	YOR298C-A	MBF1	2	4	Transcription	Transcriptional coactivator	3
21	YJL115W	ASF1	2	3	Transcription	Nucleosome assembly factor	4
22	YJL176C	SWI3	2	3	Transcription	Subunit of the SWI/SNF chromatin remodeling complex	1
23	YER064C	VHR2	2	3	Transcription	Non-essential nuclear protein	
24	YLR418C	CDC73	2	1	Transcription	Component of the Paf1p complex	
25	YMR116C	ASC1	4	4	Translation	G-protein beta subunit	3
26	YDR266C	HEL2	4	4	Translation	RING finger ubiquitin ligase (E3)	
29	YGR271W	SLH1	3	4	Translation	Putative RNA helicase related to Ski2p	
30	YML034W	SRC1	3	4	Translation	Inner nuclear membrane protein	
31	YIR001C	SGN1	2	4	Translation	Cytoplasmic RNA-binding protein	
32	YDR049W	VMS1	2	4	Translation	Component of the CCR4-NOT transcriptional complex	3
33	YML111W	BUL2	4	3	Ubiquitin	Component of the Rsp5p E3-ubiquitin ligase complex	
34	YEL013W	VAC8	4	3	Vacuole	Vacuolar membrane protein	5
35	YCL048W	SPS22	3	2	Various	Protein of unknown function	
1	YDR388W	RVS167	4	4	Cytoskeleton	Actin-associated protein	7
2	YDR129C	SAC6	4	4	Cytoskeleton	Fimbrin, actin-bundling protein	10
3	YLR337C	VRP1	4	4	Cytoskeleton	Verprolin, proline-rich actin-associated protein	7
4	YBL007C	SLA1	2	4	Cytoskeleton	Cytoskeletal protein binding protein	7
15	YLR056W	ERG3	3	4	Stress	C-5 sterol desaturase	12
27	YAL021C	CCR4	4	4	Translation	Component of the CCR4-NOT complex	12
28	YNR052C	POP2	4	4	Translation	Component of the CCR4-NOT complex	10

Sensitivity scores range from 1–4. MDR scores are derived from Parsons et al. 2004

**Fig. 2:**
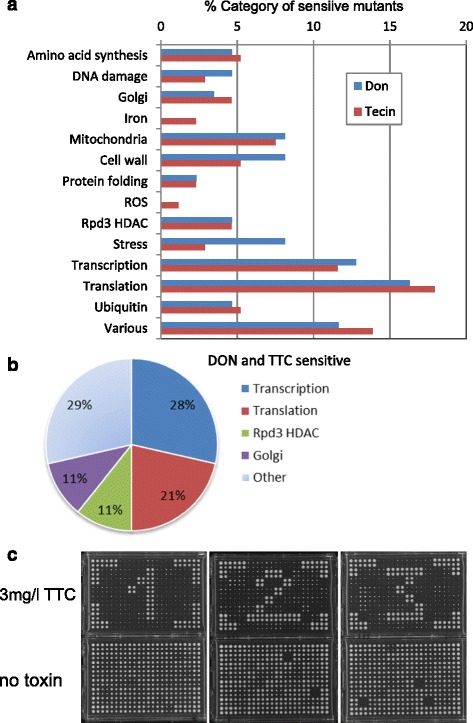
Analysis of the DON and TTC sensitive mutants. **a** Categorization of 248 mutants and their representation within the DON and TTC groups. **b** Percentage of functional categories in the 35 most sensitive strains. Strains are listed in Additional file 16: Table S2 and supplementary data files. **c** Assay plate of the verification plate of the TTC sensitive mutant library strains

To isolate the resistance promoting processes we functionally categorized the genes (Additional file 2: Data S1). Assuming that TTC and DON are primarily targeting the yeast cytoplasmic ribosome in a very similar if not identical mode of action, we supposed that a cumulative functional analysis of the obtained genes might be useful. Gene ontology (GO) annotation of all 248 identified genes as well as the 35 most sensitive mutants using the 4780 non-essential genes as background set (http://go.princeton.edu/cgi-bin/LAGO) yielded a few significant GO terms. These are including the Rpd3 histone deacetylase complex and transcription from RNA polymerase I and II promoter. Because of the few significant GO terms we manually categorized the identified genes according to their cellular function into the major aspects chromatin metabolism and transcription, amino acid synthesis, cell wall, mitochondrial functions, ion metabolism, protein folding and transport, and RNA metabolism (transport, translation, ribosome biogenesis) (Fig. 2a). Overall the proportion of DON and TTC sensitive mutants in these groups is very similar, indicating that both drugs have a similar effect on yeast physiology. Among the 35 mutants most sensitive to TTC and DON, 60 % of the respective genes are involved in transcription and translation (Fig. 2b, Table 1).

**Fig. 3:**
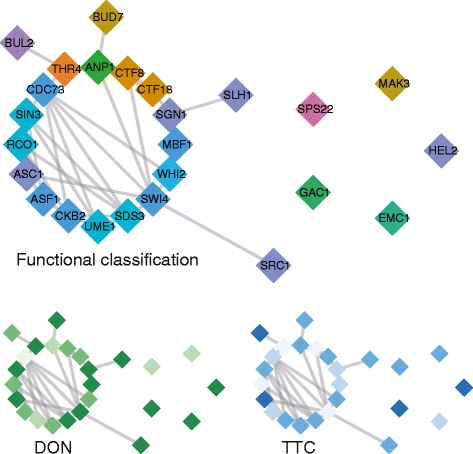
The genetic interaction networks of genes responsive to DON and TTC exhibits properties that make them significantly different from random networks. All three panels show the same network, keeping the same structure and node layout but varying the colouring in order to highlight different biological properties. In the “functional classification” panel, nodes with the same colour share similar biological properties. In the “DON” and “TTC” panel different shades of blue and green depict a response to either of the toxins, with stronger colours indicating a more pronounced response

### Network analysis of genetic interactions

To approach the basic processes encoded in such an assembly of genes with highly diverse functions we analysed their genetic interactions. Molecular functions are driven by the collaboration of genes usually represented in functional groups or pathways. To understand what these interactions are and how they are governed is a key question. One way of obtaining this information is to screen for genetic interactions, which underlie a large number of biological functions [37]. A network covering the genome-wide set of genetic interaction in yeast has been constructed [38], and allows for screening of interactions and clustered groups of interacting genes. Building upon this genetic interaction information we inferred four networks representing either a response to TTC, to DON, to either of these two (union) or both of them (intersection). To this end we used the corresponding set of genes as seeds and inferred the corresponding network from the list of genetic interactions. The resulting networks are provided as cytoscape file (Additional file 5: Data S4). At first we inspected the global clustering coefficient, which indicates the presence of local clusters within the networks that might encode for specific functionality. We found that yeast genetic interaction networks of DON or TTC responsive genes were more clustered than random networks (Additional file 6: Figure S1). This was also observed for the network of TTC or DON responsive genes. The shortest path between a pair of nodes is the minimal number of edges between them. Shorter path length might render networks more efficient at processing information than networks with another topology. This means essentially that a common connecting process exists in such a network. It has been suggested that small shortest path lengths might facilitate efficient communication between transcription factor genes [39]. The three networks (DON, TTC, DON or TTC) showed a significantly shorter average path length then their random counterparts (Additional file 7: Figure S2). The large global clustering coefficient and the short path length present typical properties of small world networks [40]. For the network diameter no such clear pattern was observed. An alternative approach for quantifying the network complexity based on topological properties is to make use of entropy-based network descriptors [41, 42]. We made use of a parametric graph entropy measure (Dehmer-entropy) which assigns a probability value to each node of a graph based on information functionals distributions [43]. These information functionals capture the structural information of a graph by considering a given probability distributions [43]. For DON, TTC, DON or TTC networks the distance measure of the sphere-based information functional distributions [43] was found to be significantly different from random networks (Additional file 8: Figure S3). This observation supports the idea that the toxin-responsive networks exhibit notable properties which render them efficient at processing information and thus collectively mediate resistance. In contrast to the topology of the DON, TTC, and DON or TTC (union) networks, the network of DON and TTC (intersection) responsive genes was not significantly different from random networks with the same number of seed genes. Genes representing the intersection of DON and TTC responsive genes might just represent indirect and pleiotropic effects of the respective mutants. One of the main functional clusters present in the intersection network (Fig. 3) was the Rpd3 histone deacetylase complex (HDAC), involved in transcriptional regulation.

### Chromatin remodelling complexes are involved in DON/TTC resistance

The network analysis points out a HDAC and a few other highly contributing genes but also shows a tight interaction over all isolated genes. Thus a common theme is likely embedded in the ensemble of the isolated genes. In the following we argue that most mutants impair directly or indirectly the regulation of cytoplasmic protein synthesis or the production and maintenance of functional ribosomes. We concentrate the discussion on groups of mutants that are highly sensitive and the respective gene products are involved in the same pathway or in the same protein complex. In the group comprising mutants in chromatin metabolism and transcription, we identified several subunits (*SDS3, SIN3, UME1, CTI6, TOD6, SAP30, RXT2)* of the RPD3L Histone deacetylase complex and *RCO1* of the RPD3S complex. The histone deacetylase Rpd3 is involved in repression of RNA Pol II driven rRNA synthesis in stationary phase [44, 45]. Moreover, loss of the Rpd3 histone deacetylase, in *rpd3* mutants or in mutants lacking *SIN3* or *SAP30*, results in a delay in rRNA processing but not in an rRNA transcriptional defect. Genetic evidence also points to rRNA posttranscriptional modification as a bottleneck in ribosome biogenesis [46]. This suggests that in Rpd3 mutants, de novo production of ribosomes might be reduced, a defect which may become more pronounced in DON and TTC treated cells due to additional inhibition of ribosomes. Alternatively, the lack of suppression of intragenic spurious transcription by the *RCO1* mutant (RPD3S complex) might generate a burden by overloading RNA surveillance mechanisms [47]. Furthermore, we identified three subunits of the SWI/SNF chromatin remodelling complex (*SNF2, SWI3, SNF6*). SWI/SNF has been reported to play a role in transcription by RNA pol I in yeast. A 2.5-fold reduction in rRNA synthesis rate was reported for *snf6Δ* cells [48]. Moreover, SWI/SNF is required for repression of RNA polymerase II-dependent transcription in the ribosomal DNA (rDNA silencing) and telomeric silencing [49]. Thus reduced or aberrantly regulated rRNA synthesis in these and other loci (*RRP8, REI1, PAF1, PIH1*) might be one underlying reason for the increased DON sensitivity. Furthermore, we detected DON sensitivity in mutants lacking the histone deacetylases *HDA1* or *HDA3* (which interact within a complex) and the alternative H2A histone H2A.Z encoded by *HTZ1*. Their influence is unclear apart from the fact that basal transcription of ribosomal genes is repressed in the corresponding mutants. Another mutant in a gene with chromatin related function was the HMG protein Nhp6A which is recruiting the FACT complex (Spt16-Pob3) and other chromatin remodelling complexes. *NHP6A* is required for transcriptional initiation of tRNA genes and assembly of the RNA polymerase II and III pre-initiation complexes. Notably, also the CCR4-Not complex has been reported to be directly involved in rRNA synthesis [50].

### Mitochondria are probably not a direct target of DON and TTC

Mitochondria have been implicated in the toxic effect of TTC [27, 28]. Genes connected to mitochondrial functions identified in this study do not seem to highlight a specific metabolic activity of mitochondria or mitochondrial translation. The relatively few mutants related to mitochondrial function (*YMC1, DIC1, MBA1, UTH1, TOM5*) and their low sensitivity does not support a model with mitochondria as major targets for both DON and TTC. The most sensitive mutant was lacking *MDM32* a mitochondrial inner membrane protein required for normal mitochondrial morphology and inheritance. *MDM32* is upstream of *PNO1* an essential gene required for 18S rRNA processing. Interference of the knock out cassette with *PNO1* expression under screening conditions cannot be excluded. The possibility remains that Pdr5-dependent drug efflux clears the cytosol of DON/TTC but not the mitochondrial matrix and is thus pronouncing effects connected to mitochondria as observed by other studies [28].

### Cell integrity and cytoskeleton are indirectly involved in DON/TTC resistance

A substantial number of DON/TTC sensitive mutants were involved in cell integrity and cell wall biosynthesis. We identified several members of the protein kinase C (PKC) signalling pathway. These are *BCK1* (MAPKKK), *SLT2* (the MAP kinase), *ZEO1* a regulator in the peripheral plasma membrane, and *SLG1* a sensor-transducer. These mutants are multidrug sensitive [22, 36] and appear to be involved in many diverse processes such as regulating maintenance of cell wall integrity, cell cycle, mitophagy and pexophagy and organization of the actin cytoskeleton. For example, Slt2 plays a role in vacuole homeostasis and actin dynamics [51]. In addition to the multifaceted pathology of these mutants, the cell integrity pathway is also involved in repression of ribosome and tRNA synthesis in secretion-defective cells [52]. In addition, we found mutants affecting cell wall integrity or functionality like 1,3-beta-D-glucan synthase Fks2 or the alpha 1–6 Mannosyltransferases *ANP1* and *HOC1*. Anp1 has been identified as conferring stress resistance in several instances [53, 54]. Cell wall synthesis is necessarily coupled to growth which in turn is dependent on biosynthesis. Alternatively, the yeast cell wall could have a protective role through drug adsorption properties. Yeast cell walls are sold as feed adjuvant with trichothecene protective activity [55]. β-glucan, is used to adsorbe DON [56, 57] and low but significant adsorption properties have been reported [56, 58]. Mutants impairing the function of the cytoskeleton are multi drug hypersensitive [36] and were found here to be highly sensitive to both DON and TTC. Their relative hypersensitivity score (MDR) [36] is listed accordingly (e.g. Table 1). We identified the interacting Actin-associated proteins Rvs167 and Rvs161 which have roles in endo- and exocytosis. Other sensitive mutants include Fimbrin (*SAC6*), Verprolin (*VRP1*) a proline-rich actin-associated protein involved in cytoskeletal organization and cytokinesis, and *SLA1* required for assembly of the cortical actin cytoskeleton. Pbs2, the MAPKK of the high osmolarity glycerol (HOG) pathway has also a function in restructuration of the cytoskeleton [59]. A possible reason why such mutants appear in many screens is the observation that rRNA levels are repressed in various mutants with defects in transport and exocytosis [60]. The unfolded protein pathway is apparently not prominently involved in TTC resistance. The *ire1* mutant was weakly sensitive to TTC and not to DON. Protein phosphatase 1 (PP1) is an enzyme clearly important for resistance against DON since we identified three genes belonging to PP1 interactors (*REG1, GIP4, GAC1)*. The catalytic subunit of PP1 (*GLC7)* is an essential gene and therefore not included in the collection and functions to promote cell integrity, mitosis, bud morphology and polarization of the actin cytoskeleton. Thus PP1 might indirectly have a profound influence on ribosome synthesis.

### Many mutants impair ribosome and ribosome quality control constituents

Genes functionally related to translation comprised several categories: ribosomal subunits (*RPL39, RPL21A, RPS27B, RPS21A, RPS23B,*), ribosome assembly (*BUD20, CMS1, RPP1A, REI1, RRP8* rRNA methyltransferase), the Cop9 signalosome components (*YJR084W, PCI8*), initiation (*SGN1, GCN3, GIS2, BUD27*) and RNA turnover. Furthermore, we identified genes involved in RNA degradation such as *LSM1,* the Ski complex (*SKI2, SKI8*), and *SLH1* (RNA helicase related to Ski2)*,* and mRNA decapping (*EDC3, PAT1*), and splicing (*BRR1, LEA2*). Interstingly, bud morphology defects can partially be attributed to impaired ribosome function. A screen for abnormal bud morphology [61] detected 111 mutants including 15 genes (significance *p* = 0.0006) here identified as DON and/or TTC sensitive. These include functions in translation (*RPS27B, RPL39, POP2, CCR4, BUD27, BUD20*), cell wall (*SLG1, PER1, FKS1, CCW12*) and cytoskeleton (*SLA1, RVS167, RVS161*). Furthermore, DON and/or TTC sensitive mutants with defects in tRNA synthesis and processing involved uridine thiolation of cytoplasmic tRNAs (*NCS6, URM1, IKI3*), a non-canonical poly(A) polymerase which catalyses polyadenylation of hypo modified tRNAs, snoRNA and rRNA precursors (*TRF5*), and a Zinc-finger protein Stp3 possibly involved in pre-tRNA splicing. An interesting connection to possible oxidative stress caused by trichothecenes is the thioredoxin peroxidase Tsa1 which acts as ribosome-associated antioxidant [62]. However, other genes with dedicated antioxidant functions were not found. Biotransformation of trichothecenes occurs partially through glutathione addition [17]. A prominent influence on DON/TTC detoxification by formation of glutathione conjugates in yeast is not supported by our data. An exception is the prion encoded by *URE2*, which can mutate to a glutathione transferase but it is involved in growth related processes as well and thus the effect of the mutant might be rather indirect.

The mutant strain with the most striking sensitivity against DON was lacking *HEL2/YDR266C*. Hel2 is an E3 ligase and has been linked genetically with the E2 Ubc4. Hel2 has initially been implicated in histone turnover [63] and connected to translation and ribosome quality control (RQC) [64, 65]. Genetic data identified *HEL2* as a high-copy-number suppressor of an allele of Mpe1, a conserved subunit of the cleavage and polyadenylation factor (CPF) [66]. Hel2 and Asc1 are factors for polypeptide quality control upstream of RQC [67, 68]. This is in line with the action of DON and TTC on early stages of translation. The RQC complex (Rqc1-Rkr1-Tae2-Cdc48-Npl4-Ufd1) is a ribosome-bound complex required for the degradation of polypeptides arising from stalled translation [67, 69]. We tested mutants lacking *RQC1* and *RKR1* and found no indication for increased DON or TTC sensitivity. Paromomycin is an aminoglycoside inducing the ribosome to bypass premature termination codons and to relax the stringency of decoding [70]. To investigate if DON/TCC toxicity is suppressed in the presence of paromomycin we determined the maximal growth rate in presence or absence of DON (12 mg/l) and a range of paromomycin concentrations (Additional file 9: Figure S4). We find a more than additive growth inhibition of the drug combination on *pdr5*Δ cells. Interestingly, we noted a higher resistance of the *hel2*Δ strain to higher doses (0.25 to 1 g/l) of paromomycin (Additional file 9: Figure S4A). Thus DON-induced stalling of ribosomes is not efficiently released by paromomycin-induced relaxation.

**Fig. 4:**
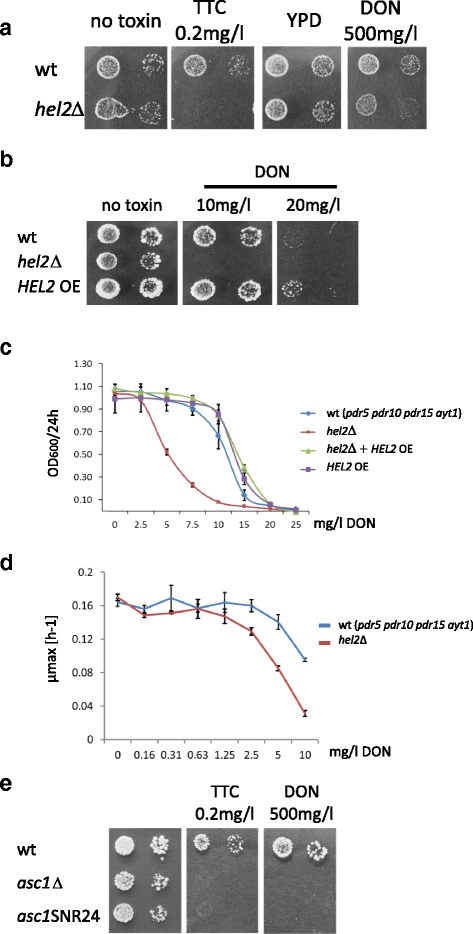
Deletion of *HEL2* confers hypersensitivity to TTC and DON. **a** Spot assay of the confirmed *hel2*Δ deletion strain in the wild type BY4741 strain background. **b** Spot assay of the *hel2*Δ deletion in a drug hypersensitive strain background (YZGN1). **c** Growth assay of the YZGN1 and YZGN1*hel2*Δ strains in presence of an *HEL2* overexpressing plasmid and the vector control. **d** Determination of growth rate of DON treated YZGN1 and YZGN1*hel2*Δ. **e** The absence of Asc1 expression causes TTC hypersensitivity but not the intron encoded snRNA

We confirmed the phenotype of the *HEL2* deletion mutant by re-testing the original deletion strain of the ordered strain collection (Fig. 4a) and by deleting the *HEL2* gene in a different strain background. For confirmation, we used a strain not related to the library strain and because of the low toxicity of DON lacking ABC transporters (Pdr5, Pdr10, Pdr15) as well as the yeast acetyl transferase Ayt1 [18]. The serial dilution tests showed a clear sensitivity of the respective *HEL2* deletion mutants to DON and TTC. Since resolving of stalled ribosomes is an activity occurring also in unperturbed cells we expected that the *HEL2* gene overexpressed under the control of the *ADH1* promoter might improve the ribosome clearing capacity. Indeed, overexpression of Hel2 conferred a slight growth advantage in presence of DON in the growth test (Fig. 4b). Growth assays using different DON concentrations (Fig. 4c) showed a marked dose-dependent decrease of growth of the mutant but also a gain of resistance by overexpression of *HEL2*. This gain is detectable in the wild type and the complemented *hel2*Δ strain (Fig. 4c). Thus Hel2 becomes limiting in DON treated cells. Also the maximal growth rate was found to be reduced in the DON treated strain relative to the wild type (Fig. 4d).

Asc1, a homolog of human RACK1 (human receptor for activated C-kinase 1) and Hel2 have been implicated in translation through polybasic peptide sequences, and may act after the ribosome has translated some distance [67, 71]. Asc1, a core component of the small (40S) ribosomal subunit which prevents frameshifting of stalled ribosomes at repeats of CGA codons is related in its effective function to Hel2 [71, 72]. Translation of CGA codon repeats is inefficient, resulting in dose-dependent stalling of ribosomes [73]. In this respect it is interesting to note the TTC sensitivity of some mutants defective in arginine biosynthesis (ARG4*, ARG5,6*) as well as membrane transporters for cationic amino acids (*RTC2, YPQ1, VBA5*). These mutants could decrease the arginine pools and thus lower the availability of the rare tRNA-Arg(UCG). Also several methionine biosynthesis genes were identified (*HOM6, MET12,13,14,22,31*). The *ASC1* gene contains an intron harbouring a snoRNA transcript unit (snoRNA U24). To distinguish between Asc1 and snoRNA dependent phenotypes we tested a mutant expressing the snoRNA U24 but not Asc1p due to a truncation of the first exon [74, 75] and found no effect from the U24 RNA (Fig. 4e). Thus the strong DON and TTC sensitivity of the mutant is due to the absence of Asc1. Asc1 is also required for general cell wall integrity and mutants are hypersensitive to iron starvation [74]. In that respect it is interesting to note that we detected genes involved in Iron metabolism (*FRA1, FET4, NFU1, MTM1*), as well as, a high-affinity copper transporter of the plasma membrane *(CTR1)* and *ARR1* a transcriptional activator involved in resistance to arsenic compounds. The Rsp5 E3-ubiquitin ligase is involved in endocytosis, membrane protein trafficking and protein quality control and thus indirectly in many cellular processes but notably also in amino acid permease sorting [76, 77]. *RSP5* is an essential gene and is therefore not present in the mutant collection but genes of two interacting proteins were identified. *BUL2* coding for a component of the Rsp5p E3-ubiquitin ligase complex and *RUP1* coding for a protein that regulates ubiquitination of Rsp5p. Taken together, we suspect that many of the isolated mutants ultimately influence cytoplasmic translation and thus cause synthetic phenotypes in the presence of DON and TTC.

### Transcription profiling reveals demand for ribosomes and histones in DON treated cells

**Fig. 5:**
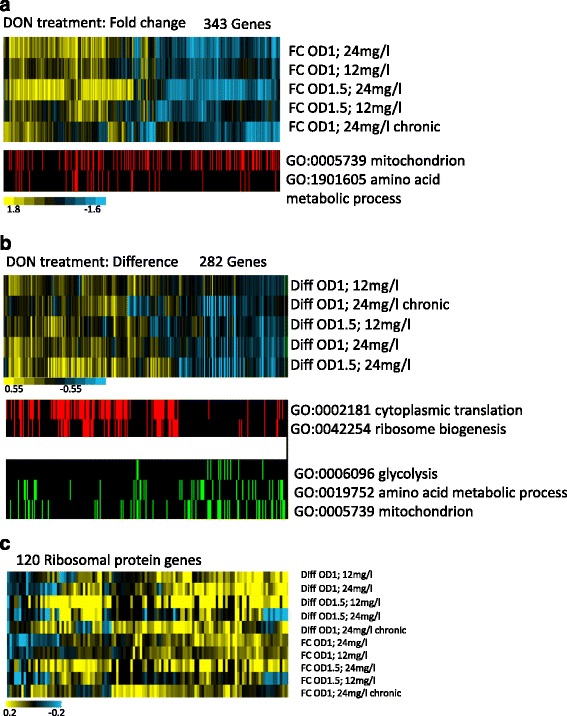
Microarray analysis of DON treated cells reveals both induced and repressed mitochondrial related genes and induced amino acid biosynthesis genes. Genes with the largest increase of transcript level (differences) are connected to cytoplasmic translation and ribosome synthesis whereas glycolysis and amino acid biosynthesis genes are repressed. Clustering of normalized and filtered expression values including enriched GO terms is indicated. **a** fold change values and **b** expression level differences. **c** Expression differences and fold change of 120 ribosomal protein genes

Gene expression pattern analysis is a powerful method to detect transcriptional responses and to deduce active signalling pathways in a given situation. Here we investigated whether specific intracellular responses are elicited by DON. We used 12 mg/l and 24 mg/l of DON which caused a slight growth delay and treated exponentially growing cells lacking Pdr5. DON was added when the cultures reached OD600 of 0.5 and cells were harvested when the cultures reached an OD600 of 1 or 1.5 and compared to untreated control cultures at the same optical density. To define the difference between short term and long term responses, we incubated cells with 24 mg/l DON overnight and re-grew them in fresh medium containing 24 mg/l DON to early exponential phase (OD600 of 0.1 to 1). All measurements were done in triplicate. Expression data were filtered and 382 genes were selected according to fold change values of above 0.5 or below−0.5 (log2) in at least one condition and analysed for GO term enrichment. We found enrichment of genes involved in mitochondrial respiratory chain function and amino acid biosynthesis, however, the distribution is almost even between the induced and repressed genes (Fig. 5a). Notably, the selected genes do not represent a specific cellular response. The gene lists and GO analysis are provided as supplementary data (Additional file 10: Data S5, Additional file 11: Data S6). We further analysed the absolute changes of signal abundance to define those transcripts which undergo the largest quantitative changes. The obtained expression level differences were normalized and arranged by hierarchical clustering (Fig. 5b). Significantly enriched GO terms connected to expression level differences revealed reduction of genes of glycolysis and amino acid biosynthesis and increase of genes involved in cytoplasmic translation. Interestingly, in this selection the levels of most ribosomal protein genes and all histone genes increased. Furthermore, expression level differences increase for most ribosomal protein genes (Fig. 5c, Additional file 12: Data S7). This possibly points to a situation caused by DON where translation is lacking behind the available carbon and energy sources. Reduced biomass production demand due to restricted growth leads to reduced glycolytic flux and a switch to respiration as energy source.

**Fig. 6:**
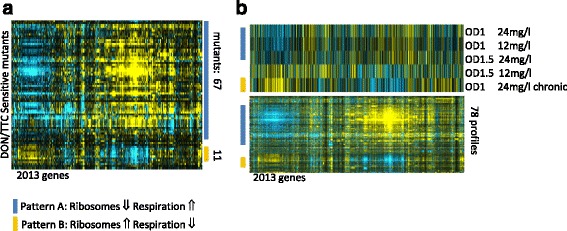
Comparative transcription pattern analysis. **a** The Microarray expression pattern of DON and TTC sensitive mutants highlights a slow growth expression pattern in many strains. **b** DON treated cells have an initial dose dependent slow growth expression pattern (Type A: blue bars) which is inverted (Type B: orange bars) at later time points. Expression values of the genes of the DON treatment are sorted in parallel to the heat map of the microarray compendium depicted below. Expression values were obtained from http://deleteome.holstegelab.nl/

Many deletion strains sensitive to DON and TTC are sensitive to many other drugs as well [36]. Notorious multidrug sensitive strains have defects in functions of vacuole, cytoskeleton and cell wall. These mutants lead to a reduction of rRNA synthesis since growth and ribosome synthesis is tightly coupled [60]. One common response to environmental perturbations is the delay of the cell cycle progression. For example, various environmental stress types trigger depolarization of the actin cytoskeleton and cause a delay of the G2 phase of the cell cycle. On the other hand, mutations or drugs that specifically impair actin organization also trigger a G2 delay [78]. Many mutants with defects in various intracellular processes lead to a delay of G2. Both delay of the cell cycle and environmental stress lead to a characteristic gene expression pattern designated "slow growth signature" [79]. This transcript pattern is characterized by reduced expression of genes involved in protein synthesis such as ribosomal protein genes and glycolysis and the increase of transcripts of genes connected to respiratory growth. We collected the available microarray data for our DON and TTC sensitive mutants [79] and aligned the normalized DON treatment expression data (Figs. 6a and Additional file 13: Figure S5). The gene lists and values generating the heat maps are provided as supplementary data (Additional file 14: Data S8, Additional file 15: Data S9). We note a high proportion of strains exhibiting the slow growth signature (Pattern A) amongst our sensitive mutants suggesting that many mutant strains are suppressing ribosome production without treatment. Exposure to DON possibly leads to further reduced availability of translation capacity in these mutants due to degradation and stalling of translating ribosomes. *CTI6, PDE2, YKR023W, VAC8, URM1, IRE1, APQ12, NTH2, URE2, VHR2, and RTG1* have the inverse transcript pattern (Pattern B). The comparison of the transcript profiles of DON-treated cells to the compendium pattern shows a slow growth signature for the early time points and a switch to the inverse pattern at later time points and during chronic exposure (Fig. 6b). Taken together this implies a reduced ribosome synthesis rate in many mutants which might be exacerbated by DON induced stalling during translation.

## Conclusion

Yeast loss of function mutants isolated here as DON or TTC sensitive collectively point to cytoplasmic translation, ribosomal efficiency and rRNA synthesis as rate limiting for fitness in the presence of these toxins. Transcriptome analysis of DON treated cells shows an increase of the transcript level of many genes encoding ribosomal protein genes and histones. This perhaps suggests that cells are preparing to enhance biosynthesis. The connector between these observations is the fact that regulation of ribosome synthesis is closely tied to regulation of cell growth rate. Synthesis of ribosomes requires a substantial proportion of the cellular biosynthetic activity. For example, synthesis of rRNA by RNA polymerase I is the major transcriptional activity of the cell, accounting for 60 % of total transcription in rapidly growing yeast cells to allow for the production of ribosomes in rapidly growing cells [80]. Yeast cells are optimizing their growth corresponding to available nutrients [80]. DON and TTC treatment is impairing the 80S ribosome and thus reducing the biosynthetic capacity of yeast cells. In this respect, our observation of increased levels of transcripts coding for ribosomal genes and histones makes sense from a yeast perspective. Relatively lower translation fidelity in presence of DON or TTC would call for increased synthesis of ribosomes to provide the appropriate biosynthetic capacity to utilize the available nutrients. Our result that overexpression of Hel2 increased resistance to DON is suggesting that ribosome fidelity is a primary bottle neck of fitness. Asc1 and Hel2 are both involved in ribosome quality control and facilitate co-translational protein ubiquitination, translation termination and possibly RNA cleavage [68]. Mutants in genes functioning at later stages of ribosome quality control such as *RKR1* and *RQC1* deletion mutants were not sensitive to TTC and DON suggesting that Asc1/Hel2 is sufficient to resolve the DON/TTC induced translation problem. Network analysis of the isolated mutants indicates a tight network, containing the Rpd3 histone deacetylase complex, centred on core intracellular processes. The overall genetic interaction networks of DON or TTC sensitive strains allowed the conclusion that a common mechanism is involved. We believe this is a cytoplasmic mechanism. Earlier work suggested a primarily mitochondrial component of Trichothecene toxicity. Microarray analysis of yeast exposed to T2 toxin showed induced oxidative stress and indications point to an effect on mitochondria [81, 82]. Similarly, a critical role of mitochondria for TTC toxicity was shown [27, 28]. Our data do not support an effect of DON and TTC on mitochondria. This may be partly rooted in the absence of Pdr5. The efflux pump might reduce the cytoplasmic pool of toxin but mitochondria might trap a substantial amount. Thus, in the *pdr5* mutant the cytoplasmic concentration might be higher and lead preferably to inhibition of cytoplasmic ribosomes. Further differences to published microarrays are also most probably treatment related [34].

The perturbation of growth promoting activities leads in many cases to reduced synthesis of components of the translation machinery [79]. Many mutants isolated here evoke a growth delay [79] also caused by adverse external parameters and many types of internal malfunctions. The pattern characterizes a reduction of ribosome synthesis and glycolysis. It is tempting to speculate that the tendency of many mutants to reduce cytoplasmic translation is exacerbated by additional inhibition by the trichothecene toxins. The response mechanisms of higher eukaryotes cells are of course much more complicated [83]. Our results might provide a useful background of functional genomics to contrast against and to highlight specific responses of plant and animal cells. A recent gene expression study of DON treated wheat demonstrated induction of amino acid biosynthesis genes and the expression of an E3 ligase in response to Fusarium [84]. Transcriptome analysis of TTC-induced expression in barley identified specific responses including ubiquitination related protein genes [85]. Notably, we identify the E3 ligase Hel2 involved in early translational quality control as possible functional analogue of the wheat Fhb1 QTL. Moreover, Hel2 was also picked in the previous screen for TTC sensitive strains [28]. Thus our study pinpoints cytoplasmic translation as the primary target of DON and TTC and we therefore suggest mechanisms enhancing translation or recovery of stalled ribosomes as main source of trichothecene resistance.

## Methods

Yeast strains used in this study are listed in Additional file 16: Table S2. The strain FY1679-28C (MATa *ura3-52 leu2Δ1 his3Δ200 trp1Δ63*) and the derived double mutant *pdr1Δ pdr3Δ* have been described [86]. YZGA280 was generated by transformation of FY1679-28C with the *pdr5Δ::hisG-URA3-hisG* disruption plasmid pYM31 [87] followed by counter selection with 5-fluoro-orotic acid (*pdr5Δ::hisG*). YZGA260 was generated by transformation of the pdr1Δ pdr3Δ mutant with plasmid pYAK7 [18] leading to *ADH1* promoter mediated overexpression of an N-terminally c-Myc-tagged Pdr5 protein. The *pdr5::hisG* disruption was also introduced into the previously described [87] near isogenic strains YALA-B1 and YALA-G4 (containing *PDR1-3*) to generate strains YZGA276 (*pdr5Δ*) and YZGA278(*PDR1-3, pdr5Δ*). YZGN1 (*pdr5Δ::loxP-his5*^*+*^*-loxP*, *pdr10Δ::hisG*, *pdr15*Δ::*loxP-NatMX-loxP*, *ayt1*Δ::*URA3*) was constructed by replacing the *TRP1* gene at the PDR5 locus with pUG27 derived loxP-his5^+^-loxP cassette [88] in strain YZGA515 (*pdr5*Δ::*TRP1, pdr10*Δ::*hisG, pdr15*Δ::*loxP-KanMX-loxP, ayt1*Δ::URA3) [18] and the derivative YZGN1 *hel2Δ::kanMX*. The synthetic genetic array analysis was performed according to the published protocol [35]. The query strain Y7092 *pdr5Δ:: NatMX* was constructed according to [35]. To introduce the *pdr5Δ* mutant allele, the ordered *S.cerevisiae* EUROSCARF mutant library was mated with the query strain Y7092 *pdr5Δ:: NatMX*. Diploid cells carrying resistance cassettes and presumably the corresponding deletion mutations were selected on kanamycin (200 mg/l) and nourseothricin (100 mg/l). The selected diploids were pinned on sporulation media supplemented with 50 mg/l of kanamycin. After 10 days at room temperature haploid segregants were selected for growth on medium containing kanamycin (200 mg/l) and lacking histidine and, followed by transfer of the resulting colonies to medium supplemented with kanamycin, nourseothricin (100 mg/l) and canavanine (50 mg/l) and lacking histidine for 3 to 5 days at 30 °C. The resulting haploid screening collection was stored at−80 °C and selected after thawing. Solid pinning was done with a ROTOR HDA (Singer Inc, GB). For screening the density was increased to 384 strains per plate and pinning was done as dilution pinning with 4 successive touches of the medium surface. Plates contained 70 to 130 mg/l DON and 3 mg/l TTC. Strains of interest were thawed, grown on YPD plates at 30 °C and then transferred to SC-His media containing kanamycin (200 mg/l) for 3 days at 30 °C. The selection of was performed on SC-His media supplemented with kanamycin (200 mg/l), nourseothricin and canavanine (50 mg/l) for 3 days. Growth rates were determined in triplicate in 96 well format in YPD. OD600 values of growth curves measured in 30 min intervals were fitted with grofit(R) to obtain μ/h values.

DON (purity >98 %) was produced and purified according to Altpeter and Posselt [89]. TTC was isolated form potato dextrose broth cultures of the Trichothecium roseum strain MA 3581 (obtained from the Austrian Center of Biological Resources) by preparative HPLC as described [90].

Network analysis: To create the DON and TTC responsive networks we used the genes that were found in our screen to be reacting to treatment with either DON or TTC as seeds. In order to filter out unspecific, general toxicity-related genes we removed genes that have been found as responsive to MDR at a score higher than 3 [36]. Interactions from the intermediate stringency level yeast genetic interaction network [38] were selected if both interaction partners were within the set of remaining seeds. The corresponding networks were then visualized using Cytoscape [91] and topological properties were calculated using the igraph, RBGL, and QuACN packages in R [92–95]. The information-theoretic distance measure was calculated by the infoTheoreticGCM method (parameter: coeff=”exp”) as provided by the QuACN package [94]. For analyzing the topological properties the largest connected component of the respective selected networks was selected. To compare the observed topological properties against random characteristics we inferred B = 1000 networks using the same number of seed genes, but replacing the actual seeds by randomly sampled genes from the genetic interaction network universe for each run.

Microarray analysis: RNA quality was checked on RNA 6000 Nano chips using a Bioanalyzer 2100 (Agilent Technologies, Palo Alto, CA, USA). Agilent’s Low Input Quick Amp Labeling Kit, one-color was used to generate fluorescent cRNA. The amplified cyanine 3-labeled cRNA samples were then purified using SV Total RNA Isolation System (Promega) and hybridized to Agilent Yeast (V2) Gene Expression Microarrays 8x15K. Microarray slides were washed and scanned with an Agilent Scanner, according to the standard protocol of the manufacturer. Information from probe features was extracted from microarray scan images using the Agilent Feature Extraction software v10.7.3. Further analyses were performed using Bioconductor [96], an open source software for the analysis of genomic data rooted in the statistical computing environment R. The raw intensities were imported into Bioconductor and further processed with the limma [97] package. Quality Controls were performed using the arrayQualityMetrics package [98]. To reduce the effects of outliers, the outlier array #4 was removed. Data was background corrected using the normexp method of limma, with an offset of 32 to stabilize low intensity variances. Normalization between arrays was performed using the quantile method, duplicate probes were averaged and a linear model was fitted. P-values were adjusted for multiple testing using the Benjamini & Hochberg method. Differences were calculated from raw intensity data followed by log transformation and normalization with cluster3 [99]. Microarray data have been deposited at GEO with the accession number GSE75462. Data from large scale expression analysis were obtained from http://deleteome.holstegelab.nl/ [79]. Visualization of cluster analysis was done with TreeView (http://jtreeview.sourceforge.net).

## Ethics

Not applicable.

## Consent to publish

Not applicable.

## Availability of supporting data

Microarray data have been deposited at GEO (http://www.ncbi.nlm.nih.gov/geo/) with the accession number GSE75462. Supporting material accompanies this manuscript. Data from large scale expression analysis (79) were obtained from http://deleteome.holstegelab.nl/.

## Additional files

Additional file 1: Table S1. Mutant strains isolated as DON (70 and 130 mg/l) and TTC (0.3 mg/l) sensitive. All strains carried an additional deletion of the *PDR5* gene. Sensitivity increases from 1–4. Abbreviations in column T: a: standard genes, b: multidrug sensitive genes, c: known unknowns. (DOCX 46 kb) Additional file 2: Data S1. Excel file including the selected 248 sensitive strains and the GO enrichment analysis. (XLSX 97 kb) Additional file 3: Data S2. Excel file including the selected 35 most sensitive strains and the GO enrichment analysis. (XLSX 58 kb) Additional file 4: Data S3. Excel file including the TTC sensitive strains also detected by Bin-Umer et al., 2014. (XLSX 12 kb) Additional file 5: Data S4. Cytoscape file of the genetic interaction network of DON and/or TTC sensitive strains based on Constanzo et al., 2008. (CYS 301 kb) Additional file 6: Figure S1. The global clustering coefficient was used as a measure of the degree of clustering in the whole network. We compared the observed value (blue line) against 1000 random seed networks for A) DON, B) TTC, C) DON and Tcin, and D) DON or TTC. (PDF 588 kb) Additional file 7: Figure S2. The average path length of the giant connected component. We compared the observed value (blue line) against 1000 random seed networks for A) DON, B) TTC, C) DON and TTC, and D) DON or TTC. (PDF 588 kb) Additional file 8: Figure S3. Assessment of topological properties based on a Dehmer entropy measure. We compared the observed value (blue line) against 1000 random seed networks for A) DON, B) TTC, C) DON and Tcin, and D) DON or TTC. (PDF 588 kb) Additional file 9: Figure S4. Combinatorial effect of paromomycin and DON. A) Maximum growth rates determined from fitting of growth curves with grofit(R). The amount of paromomycin and the inclusion of DON are indicated. Growth was recorded in triplicates in 96 well plates in YPD in 30 min intervals. Standard deviations are indicated. B) Expected combinatorial effect of DON and paromomycin for the BY4641 *pdr5*Δ strain. The expected value for a multiplicative effect on growth was set to 100 %. Standard deviations are indicated. (PDF 589 kb) Additional file 10: Data S5. Values to visualize the heat map of Fig. 5a. (TXT 26 kb) Additional file 11: Data S6. Values to visualize the heat map of Fig. 5b. (TXT 21 kb) Additional file 12: Data S7. Values to visualize the heat map of Fig. 5c. (TXT 13 kb) Additional file 13: Figure S5. Microarray data derived from O’Duibhir et al. (Molecular systems biology 2014, 10:732.) of genes identified as conferring DON and TTC resistance. Data to visualize the heat map is included in Additional file 12: Data S7. (PDF 588 kb) Additional file 14: Data S8. Values to visualize the heat map of Fig. 6a. (TXT 3521 kb) Additional file 15: Data S9. Values to visualize the heat map of Fig. 6b. (TXT 2022 kb) Additional file 16: Table S2. Strains used in this study. (DOCX 17 kb)

## Abbreviations

DON: deoxynivalenol
GO: Gene ontology
pol: polymerase
TTC: thrichothecin
