# Supplementary material for: Ribosome quality control is a central protection mechanism for yeast exposed to deoxynivalenol and trichothecin
Source: BMC Genomics. 2016 Jun 1;17:417. doi: 10.1186/s12864-016-2718-y (PMC4888481; doi:10.1186/s12864-016-2718-y)
Supplement: Additional file 1: Table S1. — Mutant strains isolated as DON (70 and 130 mg/l) and TTC (0.3 mg/l) sensitive. All strains carried an additional deletion of the PDR5 gene. Sensitivity increases from 1–4. Abbreviations in column T: a: standard genes, b: multidrug sensitive genes, c: known unknowns. (DOCX 46 kb) [file 12864_2016_2718_MOESM1_ESM.docx]

**Supplementary Table 1**: Mutant strains isolated as DON (70 and 130mg/l) and TTC (0.3mg/l) sensitive. All strains carried an additional deletion of the *PDR5* gene. Sensitivity increases from 1-4. Abbreviations in column T: a: standard genes, b: multidrug sensitive genes, c: known unknowns

|  | **YORF** | **NAME** | **Description** | **DON** | **TTC** | **Category** | T |
| --- | --- | --- | --- | --- | --- | --- | --- |
| 1 | YPL038W | MET31 | Zinc-finger DNA-binding transcription factor | 3 |  | Amino acid biosysnthesis | a |
| 2 | YCR053W | THR4 | Threonine synthase | 1 | 4 | Amino acid biosysnthesis | a |
| 3 | YGL125W | MET13 | Major isozyme of methylenetetrahydrofolate reductase |  | 4 | Amino acid biosysnthesis | a |
| 4 | YHR025W | THR1 | Homoserine kinase |  | 4 | Amino acid biosysnthesis | a |
| 5 | YJR139C | HOM6 | Homoserine dehydrogenase (L-homoserine:NADP oxidoreductase) |  | 4 | Amino acid biosysnthesis | a |
| 6 | YOL064C | MET22 | Bisphosphate-3'-nucleotidase |  | 4 | Amino acid biosysnthesis | a |
| 7 | YHR018C | ARG4 | Argininosuccinate lyase |  | 3 | Amino acid biosysnthesis | a |
| 8 | YER069W | ARG5,6 | Acetylglutamate kinase and N-acetyl-gamma-glutamyl-phosphate reductase |  | 3 | Amino acid biosysnthesis | a |
| 9 | YKL001C | MET14 | Adenylylsulfate kinase |  | 3 | Amino acid biosysnthesis | a |
| 10 | YBR069C | TAT1 | Amino acid transporter for valine, leucine, isoleucine, and tyrosine |  | 2 | Amino acid biosysnthesis | a |
| 11 | YBR147W | RTC2 | Putative vacuolar membrane transporter for cationic amino acids | 1 |  | Amino acid biosysnthesis | a |
| 12 | YOL092W | YPQ1 | Putative vacuolar membrane transporter for cationic amino acids | 1 |  | Amino acid biosysnthesis | a |
| 13 | YLR342W | FKS1 | Catalytic subunit of 1,3-beta-D-glucan synthase | 2 |  | Cell wall | a |
| 14 | YOL109W | ZEO1 | Peripheral membrane protein of the plasma membrane | 1 |  | Cell wall | a |
| 15 | YLR110C | CCW12 | Cell wall mannoprotein |  | 3 | Cell wall | a |
| 16 | YGR229C | SMI1 | Protein involved in the regulation of cell wall synthesis |  | 3 | Cell wall | a |
| 17 | YJL128C | PBS2 | MAP kinase kinase of the HOG signaling pathway |  | 3 | Cell wall | a |
| 18 | YOR008C | SLG1 | Sensor-transducer of the stress-activated PKC1-MPK1 kinase pathway |  | 2 | Cell wall | a |
| 19 | YER011W | TIR1 | Cell wall mannoprotein | 2 |  | Cell wall | a |
| 20 | YBR162C | TOS1 | Covalently-bound cell wall protein of unknown function | 1 |  | Cell wall | a |
| 21 | YEL036C | ANP1 | Subunit of the alpha-1,6 mannosyltransferase complex | 3 | 2 | Cell wall | a |
| 22 | YJR075W | HOC1 | Alpha-1,6-mannosyltransferase |  | 4 | Cell wall | a |
| 23 | YCR044C | PER1 | Protein of the endoplasmic reticulum | 1 |  | Cell wall | a |
| 24 | YPR171W | BSP1 | Adapter that links synaptojanins to the cortical actin cytoskeleton | 1 |  | Cytoskeleton | a |
| 25 | YER155C | BEM2 | Rho GTPase activating protein (RhoGAP) |  | 3 | Cytoskeleton | a |
| 26 | YMR078C | CTF18 | Subunit of a complex with Ctf8p | 3 | 3 | DNA damage | a |
| 27 | YHR191C | CTF8 | Subunit of a complex with Ctf18p | 1 | 3 | DNA damage | a |
| 28 | YLR032W | RAD5 | DNA helicase/Ubiquitin ligase | 1 |  | DNA damage | a |
| 29 | YOL090W | MSH2 | Protein that binds to DNA mismatches | 1 |  | DNA damage | a |
| 30 | YOR189W | IES4 | Component of the INO80 chromatiin remodeling complex |  | 4 | DNA damage | a |
| 31 | YDL101C | DUN1 | Cell-cycle checkpoint serine-threonine kinase |  | 3 | DNA damage | a |
| 32 | YEL037C | RAD23 | Protein with ubiquitin-like N terminus |  | 3 | DNA damage | a |
| 33 | YOR300W | BUD7 | Member of the ChAPs family (Chs5p-Arf1p-binding proteins) | 3 | 4 | Golgi | a |
| 34 | YPR051W | MAK3 | Catalytic subunit of the NatC type N-terminal acetyltransferase | 3 | 2 | Golgi | a |
| 35 | YLR261C | YPT6 | Rab family GTPase | 3 |  | Golgi | a |
| 36 | YFL048C | EMP47 | Integral membrane component of ER-derived COPII-coated vesicles |  | 4 | Golgi | a |
| 37 | YGL167C | PMR1 | High affinity Ca2+/Mn2+ P-type ATPase |  | 4 | Golgi | a |
| 38 | YGL199C | YIP4 | Protein that interacts with Rab GTPases |  | 4 | Golgi | a |
| 39 | YKL063C | YKL063C | Putative protein of unknown function |  | 3 | Golgi | a |
| 40 | YGL223C | COG1 | Essential component of the conserved oligomeric Golgi complex |  | 3 | Golgi | a |
| 41 | YHR181W | SVP26 | Integral membrane protein of the early Golgi apparatus and ER |  | 3 | Golgi | a |
| 42 | YLL029W | FRA1 | Protein involved in negative regulation of iron regulon transcription |  | 4 | iron_metabolism | a |
| 43 | YMR319C | FET4 | Low-affinity Fe(II) transporter of the plasma membrane |  | 3 | iron_metabolism | a |
| 44 | YKL040C | NFU1 | Protein involved in iron metabolism in mitochondria |  | 3 | iron_metabolism | a |
| 45 | YGR257C | MTM1 | Mitochondrial protein of the mitochondrial carrier family |  | 2 | iron_metabolism | a |
| 46 | YPR058W | YMC1 | Putative mitochondrial inner membrane transporter |  | 4 | Mitochondria | a |
| 47 | YOR147W | MDM32 | Mitochondrial inner membrane protein with similarity to Mdm31p | 4 | 4 | Mitochondria | a |
| 48 | YLL001W | DNM1 | Dynamin-related GTPase involved in mitochondrial organization | 2 |  | Mitochondria | a |
| 49 | YDL085W | NDE2 | Mitochondrial external NADH dehydrogenase | 1 |  | Mitochondria | a |
| 50 | YAR035W | YAT1 | Outer mitochondrial carnitine acetyltransferase |  | 4 | Mitochondria | a |
| 51 | YGL218W | MDM34 | Mitochondrial component of the ERMES complex |  | 2 | Mitochondria | a |
| 52 | YMR072W | ABF2 | Mitochondrial DNA-binding protein | 2 |  | Mitochondria | a |
| 53 | YJL102W | MEF2 | Mitochondrial elongation factor involved in translational elongation | 2 |  | Mitochondria | a |
| 54 | YLR252W | SYM1 | Protein required for ethanol metabolism |  | 4 | Mitochondria | a |
| 55 | YPL271W | ATP15 | Epsilon subunit of the F1 sector of mitochondrial F1F0 ATP synthase |  | 3 | Mitochondria | a |
| 56 | YDR150W | NUM1 | Protein required for nuclear migration |  | 3 | Mitochondria | a |
| 57 | YGR174C | CBP4 | Mitochondrial protein required for assembly of cytochrome bc1 complex |  | 2 | Mitochondria | a |
| 58 | YOL008W | COQ10 | Coenzyme Q (ubiquinone) binding protein |  | 2 | Mitochondria | a |
| 59 | YLR349W | DIC1 | Mitochondrial dicarboxylate carrier |  | 2 | Mitochondria | a |
| 60 | YKR023W | YKR023W | Putative protein of unknown function |  | 4 | Mitochondria | a |
| 61 | YKR042W | UTH1 | Mitochondrial inner membrane protein |  | 4 | Mitochondria | a |
| 62 | YPR133W-A | TOM5 | Component of the TOM (translocase of outer membrane) complex |  | 2 | Mitochondria | a |
| 63 | YMR024W | MRPL3 | Mitochondrial ribosomal protein of the large subunit | 1 |  | Mitochondria | a |
| 64 | YBR185C | MBA1 | Membrane-associated mitochondrial ribosome receptor |  | 3 | Mitochondria | a |
| 65 | YER087W | AIM10 | Protein with similarity to tRNA synthetases | 1 |  | Mitochondria | a |
| 66 | YHR046C | INM1 | Inositol monophosphatase | 1 |  | PIP | a |
| 67 | YGR223C | HSV2 | Phosphatidylinositol 3,5-bisphosphate-binding protein |  | 4 | PIP | a |
| 68 | YDR028C | REG1 | Regulatory subunit of type 1 protein phosphatase Glc7p |  | 2 | PP1 | a |
| 69 | YAL031C | GIP4 | Cytoplasmic protein that regulates protein phosphatase 1 Glc7p |  | 2 | PP1 | a |
| 70 | YOR178C | GAC1 | Regulatory subunit for Glc7p type-1 protein phosphatase (PP1) | 4 | 4 | PP1 | a |
| 71 | YCL046W | EMC1 | Member of conserved endoplasmic reticulum membrane complex | 2 | 4 | Protein_folding | a |
| 72 | YGR078C | PAC10 | Part of the heteromeric co-chaperone GimC/prefoldin complex |  | 4 | Protein_folding | a |
| 73 | YOR007C | SGT2 | Glutamine-rich cytoplasmic cochaperone |  | 3 | Protein_folding | a |
| 74 | YHR034C | PIH1 | Component of the conserved R2TP complex (Rvb1-Rvb2-Tah1-Pih1) |  | 2 | Protein_folding | a |
| 75 | YHR079C | IRE1 | Serine-threonine kinase and endoribonuclease | 2 |  | Protein_folding | a |
| 76 | YGR234W | YHB1 | Nitric oxide oxidoreductase |  | 3 | ROS | a |
| 77 | YHR183W | GND1 | 6-phosphogluconate dehydrogenase (decarboxylating) |  | 4 | ROS | a |
| 78 | YIL084C | SDS3 | Component of the Rpd3L histone deacetylase complex | 3 | 4 | Rpd3 HDAC | a |
| 79 | YOL004W | SIN3 | Component of both the Rpd3S and Rpd3L histone deacetylase complexes | 3 | 4 | Rpd3 HDAC | a |
| 80 | YPL139C | UME1 | Component of both the Rpd3S and Rpd3L histone deacetylase complexes | 3 | 3 | Rpd3 HDAC | a |
| 81 | YPL182C | CTI6 | Component of the Rpd3L histone deacetylase complex |  | 4 | Rpd3 HDAC | a |
| 82 | YBL054W | TOD6 | PAC motif binding protein involved in rRNA and ribosome biogenesis |  | 4 | Rpd3 HDAC | a |
| 83 | YMR263W | SAP30 | Component of Rpd3L histone deacetylase complex |  | 3 | Rpd3 HDAC | a |
| 84 | YBR095C | RXT2 | Component of the histone deacetylase Rpd3L complex |  | 3 | Rpd3 HDAC | a |
| 85 | YMR075W | RCO1 | Essential component of the Rpd3S histone deacetylase complex | 1 | 3 | Rpd3 HDAC | a |
| 86 | YOR043W | WHI2 | Protein required for full activation of the general stress response | 3 | 3 | Stress | a |
| 87 | YDL022W | GPD1 | NAD-dependent glycerol-3-phosphate dehydrogenase | 2 |  | Stress | a |
| 88 | YKR105C | VBA5 | Plasma membrane protein of the Major Facilitator Superfamily (MFS) |  | 4 | Amino acid biosysnthesis | a |
| 89 | YPR192W | AQY1 | Spore-specific water channel |  | 4 | Stress | a |
| 90 | YKR007W | MEH1 | Component of the EGO and GSE complexes | 1 |  | Stress | a |
| 91 | YBR001C | NTH2 | Putative neutral trehalase, required for thermotolerance | 2 |  | Stress | a |
| 92 | YOL067C | RTG1 | Transcription factor (bHLH) involved in interorganelle communication | 2 |  | Stress | a |
| 93 | YIL013C | PDR11 | ATP-binding cassette (ABC) transporter | 1 |  | Stress | a |
| 94 | YEL013W | VAC8 | Phosphorylated and palmitoylated vacuolar membrane protein | 4 | 3 | Stress | a |
| 95 | YNL242W | ATG2 | Peripheral membrane protein required for autophagic vesicle formation |  | 3 | Stress | a |
| 96 | YBR103W | SIF2 | WD40 repeat-containing subunit of Set3C histone deacetylase complex |  | 4 | Transcription | a |
| 97 | YGL025C | PGD1 | Subunit of the RNA polymerase II mediator complex | 3 | 3 | Transcription | a |
| 98 | YOR039W | CKB2 | Beta' regulatory subunit of casein kinase 2 (CK2) | 3 | 2 | Transcription | a |
| 99 | YEL007W | MIT1 | Transcriptional regulator of pseudohyphal growth | 3 | 2 | Transcription | a |
| 100 | YOR298C-A | MBF1 | Transcriptional coactivator | 2 | 4 | Transcription | a |
| 101 | YJL115W | ASF1 | Nucleosome assembly factor | 2 | 3 | Transcription | a |
| 102 | YER064C | VHR2 | Non-essential nuclear protein | 2 | 3 | Transcription | a |
| 103 | YLR418C | CDC73 | Component of the Paf1p complex | 2 | 1 | Transcription | a |
| 104 | YBR279W | PAF1 | Component of the Paf1p complex involved in transcription elongation | 1 |  | Transcription | a |
| 105 | YNL021W | HDA1 | Putative catalytic subunit of a class II histone deacetylase complex | 2 |  | Transcription | a |
| 106 | YPR179C | HDA3 | Subunit of the HDA1 histone deacetylase complex |  | 2 | Transcription | a |
| 107 | YPR065W | ROX1 | Heme-dependent repressor of hypoxic genes |  | 4 | Transcription | a |
| 108 | YGL070C | RPB9 | RNA polymerase II subunit B12.6 |  | 4 | Transcription | a |
| 109 | YOL012C | HTZ1 | Histone variant H2AZ |  | 4 | Transcription | a |
| 110 | YHR178W | STB5 | Transcription factor |  | 3 | Transcription | a |
| 111 | YNL136W | EAF7 | Subunit of the NuA4 histone acetyltransferase complex |  | 3 | Transcription | a |
| 112 | YDL005C | MED2 | Subunit of the RNA polymerase II mediator complex |  | 3 | Transcription | a |
| 113 | YPR053C | NHP6A | High-mobility group (HMG) protein |  | 3 | Transcription | a |
| 114 | YJL175W | SWI3 | Subunit of the SWI/SNF chromatin remodeling complex | 2 | 3 | Transcription | a |
| 115 | YHL025W | SNF6 | Subunit of the SWI/SNF chromatin remodeling complex |  | 3 | Transcription | a |
| 116 | YKR077W | MSA2 | Putative transcriptional activator |  | 4 | Transcription | a |
| 117 | YER111C | SWI4 | DNA binding component of the SBF complex (Swi4p-Swi6p) | 1 | 4 | Transcription | a |
| 118 | YIR003W | AIM21 | Protein of unknown function |  | 3 | Transcription | a |
| 119 | YMR116C | ASC1 | ortholog of RACK1 that inhibits translation | 4 | 4 | Translation | a |
| 120 | YDR266C | HEL2 | RING finger ubiquitin ligase (E3) | 4 | 4 | Translation | a |
| 121 | YJL189W | RPL39 | Ribosomal 60S subunit protein L39 | 4 |  | Translation | a |
| 122 | YDR048C | VMS1 | Component of the CCR4-NOT transcriptional complex | 2 | 4 | Translation | a |
| 123 | YGR271W | SLH1 | Putative RNA helicase related to Ski2p | 3 | 4 | Translation | a |
| 124 | YML034W | SRC1 | Inner nuclear membrane protein | 3 | 4 | Translation | a |
| 125 | YIR001C | SGN1 | Cytoplasmic RNA-binding protein | 2 | 4 | Translation | a |
| 126 | YLR374C | STP3 | Zinc-finger protein of unknown function | 1 |  | Translation | a |
| 127 | YPR096C | YPR096C | Protein of unknown function | 1 |  | Translation | a |
| 128 | YBR191W | RPL21A | Ribosomal 60S subunit protein L21A | 1 |  | Translation | a |
| 129 | YHR021C | RPS27B | Protein component of the small (40S) ribosomal subunit | 1 |  | Translation | a |
| 130 | YMR040W | YET2 | Protein of unknown function that may interact with ribosomes | 1 |  | Translation | a |
| 131 | YPR057W | BRR1 | snRNP protein component of spliceosomal snRNPs |  | 4 | Translation | a |
| 132 | YKR057W | RPS21A | Protein component of the small (40S) ribosomal subunit |  | 4 | Translation | a |
| 133 | YGL211W | NCS6 | Protein required for uridine thiolation of Gln, Lys, and Glu tRNAs |  | 4 | Translation | a |
| 134 | YPL178W | CBC2 | Small subunit of the heterodimeric cap binding complex with Sto1p |  | 4 | Translation | a |
| 135 | YLR003C | CMS1 | Putative subunit of the 90S preribosome processome complex |  | 4 | Translation | a |
| 136 | YDL081C | RPP1A | Ribosomal stalk protein P1 alpha |  | 4 | Translation | a |
| 137 | YNL299W | TRF5 | Non-canonical poly(A) polymerase |  | 4 | Translation | a |
| 138 | YLR074C | BUD20 | C2H2-type zinc finger protein required for ribosome assembly |  | 3 | Translation | a |
| 139 | YLR384C | IKI3 | Subunit of Elongator complex |  | 3 | Translation | a |
| 140 | YJL028W | YJL028W | Protein of unknown function |  | 3 | Translation | a |
| 141 | YFL023W | BUD27 | Unconventional prefoldin protein involved in translation initiation |  | 3 | Translation | a |
| 142 | YEL015W | EDC3 | Non-essential conserved protein with a role in mRNA decapping |  | 3 | Translation | a |
| 143 | YNL255C | GIS2 | Translational activator for mRNAs with internal ribosome entry sites |  | 3 | Translation | a |
| 144 | YBR267W | REI1 | Cytoplasmic pre-60S factor |  | 3 | Translation | a |
| 145 | YPR132W | RPS23B | Ribosomal protein 28 (rp28) of the small (40S) ribosomal subunit |  | 3 | Translation | a |
| 146 | YLR398C | SKI2 | Ski complex component and putative RNA helicase |  | 3 | Translation | a |
| 147 | YGL213C | SKI8 | Ski complex component and WD-repeat protein |  | 3 | Translation | a |
| 148 | YML028W | TSA1 | Thioredoxin peroxidase |  | 3 | Translation | a |
| 149 | YDR083W | RRP8 | Nucleolar S-adenosylmethionine-dependent rRNA methyltransferase |  | 2 | Translation | a |
| 150 | YIL040W | APQ12 | Protein required for nuclear envelope morphology |  | 2 | Translation | a |
| 151 | YKR026C | GCN3 | Alpha subunit of translation initiation factor eIF2B |  | 1 | Translation | a |
| 152 | YPL213W | LEA1 | Component of U2 snRNP complex |  | 4 | Translation | a |
| 153 | YIL071C | PCI8 | Possible shared subunit of Cop9 signalosome (CSN) and eIF3 | 1 |  | Ubiquitin_metabolism | a |
| 154 | YJR084W | YJR084W | Protein that forms a complex with Thp3p |  | 3 | Ubiquitin_metabolism | a |
| 155 | YML111W | BUL2 | Component of the Rsp5p E3-ubiquitin ligase complex | 4 | 3 | Ubiquitin_metabolism | a |
| 156 | YLR416C | VPS36 | Component of the ESCRT-II complex | 2 |  | Ubiquitin_metabolism | a |
| 157 | YPL065W | VPS28 | Component of the ESCRT-I complex | 2 |  | Ubiquitin_metabolism | a |
| 158 | YJL211C | PEX2 | RING-finger peroxin and E3 ubiquitin ligase | 1 |  | Ubiquitin_metabolism | a |
| 159 | YDL122W | UBP1 | Ubiquitin-specific protease |  | 4 | Ubiquitin_metabolism | a |
| 160 | YMR119W | ASI1 | Subunit of the nuclear inner membrane Asi ubiquitin ligase complex |  | 4 | Ubiquitin_metabolism | a |
| 161 | YGL110C | CUE3 | Protein of unknown function |  | 4 | Ubiquitin_metabolism | a |
| 162 | YOR138C | RUP1 | Protein that regulates ubiquitination of Rsp5p |  | 3 | Ubiquitin_metabolism | a |
| 163 | YBR082C | UBC4 | Ubiquitin-conjugating enzyme (E2) |  | 3 | Ubiquitin_metabolism | a |
| 164 | YPR164W | MMS1 | Subunit of E3 ubiquitin ligase complex involved in replication repair |  | 3 | Ubiquitin_metabolism | a |
| 165 | YBR058C | UBP14 | Ubiquitin-specific protease |  | 3 | Ubiquitin_metabolism | a |
| 166 | YIL008W | URM1 | Ubiquitin-like protein involved in thiolation of cytoplasmic tRNAs |  | 3 | Ubiquitin_metabolism | a |
| 167 | YNL015W | PBI2 | Cytosolic inhibitor of vacuolar proteinase B (PRB1) |  | 4 | Various | a |
| 168 | YCL048W | SPS22 | Protein of unknown function | 3 | 2 | Various | a |
| 169 | YHR153C | SPO16 | Meiosis-specific protein involved in synaptonemal complex assembly |  | 3 | Various | a |
| 170 | YIL049W | DFG10 | Probable polyprenol reductase | 3 | 2 | Various | a |
| 171 | YJL051W | IRC8 | Bud tip localized protein of unknown function | 3 |  | Various | a |
| 172 | YMR205C | PFK2 | Beta subunit of heterooctameric phosphofructokinase | 2 |  | Various | a |
| 173 | YHL003C | LAG1 | Ceramide synthase component | 2 |  | Various | a |
| 174 | YGR288W | MAL13 | MAL-activator protein | 2 |  | Various | a |
| 175 | YKL221W | MCH2 | Protein with similarity to mammalian monocarboxylate permeases | 2 |  | Various | a |
| 176 | YGR242W | YAP1802 | Protein of the AP180 family, involved in clathrin cage assembly | 1 | 3 | Various | a |
| 177 | YPL023C | MET12 | Protein with MTHFR activity in vitro | 1 |  | Various | a |
| 178 | YGL221C | NIF3 | Protein of unknown function | 1 |  | Various | a |
| 179 | YNL298W | CLA4 | Cdc42p-activated signal transducing kinase |  | 4 | Various | a |
| 180 | YJL204C | RCY1 | F-box protein involved in recycling endocytosed proteins |  | 4 | Various | a |
| 181 | YOR360C | PDE2 | High-affinity cyclic AMP phosphodiesterase |  | 4 | Various | a |
| 182 | YFR046C | CNN1 | Kinetochore protein |  | 4 | Various | a |
| 183 | YDR507C | GIN4 | Protein kinase involved in bud growth and assembly of the septin ring |  | 4 | Various | a |
| 184 | YGR144W | THI4 | Thiazole synthase |  | 4 | Various | a |
| 185 | YPL074W | YTA6 | Putative ATPase of the CDC48/PAS1/SEC18 (AAA) family |  | 4 | Various | a |
| 186 | YPR039W | TIP41 | Protein that interacts with Tap42p, which regulates PP2A |  | 3 | Various | a |
| 187 | YPR123C | CTR1 | High-affinity copper transporter of the plasma membrane |  | 3 | Various | a |
| 188 | YLL007C | LMO1 | Homolog of mammalian ELMO (Engulfment and celL MOtility) |  | 3 | Various | a |
| 189 | YJR074W | MOG1 | Conserved nuclear protein that interacts with GTP-Gsp1p |  | 3 | Various | a |
| 190 | YLR351C | NIT3 | Nit protein |  | 3 | Various | a |
| 191 | YLR079W | SIC1 | Cyclin-dependent kinase inhibitor (CKI) |  | 3 | Various | a |
| 192 | YPR121W | THI22 | Protein with similarity to hydroxymethylpyrimidine phosphate kinases |  | 3 | Various | a |
| 193 | YPR199C | ARR1 | Transcriptional activator of the basic leucine zipper (bZIP) family |  | 2 | Transcription | a |
| 194 | YPL241C | CIN2 | GTPase-activating protein (GAP) for Cin4p |  | 2 | Various | a |
| 195 | YCL058C | FYV5 | Protein involved in regulation of the mating pathway |  | 2 | Various | a |
| 196 | YJL135W | LCB3 | Long-chain base-1-phosphate phosphatase |  | 2 | Various | a |
| 197 | YKR003W | OSH6 | Member of an oxysterol-binding protein family |  | 1 | Various | a |
| 198 | YJL095W | BCK1 | MAPKKK acting in the protein kinase C signaling pathway |  | 4 | Cell wall | b |
| 199 | YHR030C | SLT2 | Serine/threonine MAP kinase |  | 3 | Cell wall | b |
| 200 | YDR388W | RVS167 | Actin-associated protein with roles in endocytosis and exocytosis | 4 | 4 | Cytoskeleton | b |
| 201 | YDR129C | SAC6 | Fimbrin, actin-bundling protein | 4 | 4 | Cytoskeleton | b |
| 202 | YLR337C | VRP1 | Verprolin, proline-rich actin-associated protein | 4 | 4 | Cytoskeleton | b |
| 203 | YBL007C | SLA1 | Cytoskeletal protein binding protein | 2 | 4 | Cytoskeleton | b |
| 204 | YCR009C | RVS161 | Amphiphysin-like lipid raft protein |  | 4 | Cytoskeleton | b |
| 205 | YLR056W | ERG3 | C-5 sterol desaturase | 3 | 4 | Stress | b |
| 206 | YNL229C | URE2 | Nitrogen catabolite repression transcriptional regulator |  | 4 | Transcription | b |
| 207 | YOR290C | SNF2 | Catalytic subunit of the SWI/SNF chromatin remodeling complex |  | 4 | Transcription | b |
| 208 | YCR077C | PAT1 | Deadenylation-dependent mRNA-decapping factor |  | 3 | Translation | b |
| 209 | YAL021C | CCR4 | Component of the CCR4-NOT transcriptional complex | 4 | 4 | Translation | b |
| 210 | YNR052C | POP2 | RNase of the DEDD superfamily | 4 | 4 | Translation | b |
| 211 | YEL051W | VMA8 | Subunit D of the V1 peripheral membrane domain of V-ATPase | 2 |  | Vacuole | b |
| 212 | YPL234C | VMA11 | Vacuolar ATPase V0 domain subunit c' |  | 4 | Vacuole | b |
| 213 | YPR036W | VMA13 | Subunit H of the V1 peripheral membrane domain of V-ATPase |  | 4 | Vacuole | b |
| 214 | YHR060W | VMA22 | Protein that is required for vacuolar H+-ATPase (V-ATPase) function |  | 2 | Vacuole | b |
| 215 | YJL124C | LSM1 | Lsm (Like Sm) protein |  | 3 | Various | b |
| 216 | YGR188C | BUB1 | Protein kinase involved in the cell cycle checkpoint into anaphase |  | 3 | Various | b |
| 217 | YGR092W | DBF2 | Ser/Thr kinase involved in transcription and stress response |  | 3 | Various | b |
| 218 | YOR306C | MCH5 | Plasma membrane riboflavin transporter |  | 2 | Various | b |
| 219 | YCL036W | GFD2 | Protein of unknown function |  | 3 | known_unknowns | c |
| 220 | YLR177W | YLR177W | Putative protein of unknown function |  | 3 | known_unknowns | c |
| 221 | YFL051C | YFL051C | Putative protein of unknown function | 2 |  | known_unknowns | c |
| 222 | YBR144C | YBR144C | Dubious open reading frame | 1 |  | known_unknowns | c |
| 223 | YLR402W | YLR402W | Dubious open reading frame |  | 3 | known_unknowns | c |
| 224 | YKL121W | DGR2 | Protein of unknown function |  | 3 | known_unknowns | c |
| 225 | YLR257W | YLR257W | Protein of unknown function |  | 3 | known_unknowns | c |
| 226 | YLR012C | YLR012C | Putative protein of unknown function | 3 |  | known_unknowns | c |
| 227 | YHL019C | APM2 | Protein of unknown function |  | 2 | known_unknowns | c |
| 228 | YMR126C | DLT1 | Protein of unknown function |  | 2 | known_unknowns | c |
| 229 | YHR112C | YHR112C | Protein of unknown function |  | 2 | known_unknowns | c |
| 230 | YPR027C | YPR027C | Putative protein of unknown function |  | 4 | known_unknowns | c |
| 231 | YKR045C | YKR045C | Putative protein of unknown function |  | 4 | known_unknowns | c |
| 232 | YEL001C | IRC22 | Putative protein of unknown function |  | 4 | known_unknowns | c |
| 233 | YLR271W | YLR271W | Putative protein of unknown function |  | 4 | known_unknowns | c |
| 234 | YKL075C | YKL075C | Putative protein of unknown function |  | 4 | known_unknowns | c |
| 235 | YLR287C | YLR287C | Putative protein of unknown function |  | 4 | known_unknowns | c |
| 236 | YLR125W | YLR125W | Putative protein of unknown function |  | 4 | known_unknowns | c |
| 237 | YBR139W | YBR139W | Putative serine type carboxypeptidase |  | 4 | known_unknowns | c |
| 238 | YFR045W | YFR045W | Putative mitochondrial transport protein |  | 2 | known_unknowns | c |
| 239 | YBR219C | YBR219C | Putative protein of unknown function |  | 2 | known_unknowns | c |
| 240 | YDL057W | YDL057W | Putative protein of unknown function |  | 2 | known_unknowns | c |
| 241 | YJR115W | YJR115W | Putative protein of unknown function |  | 2 | known_unknowns | c |
| 242 | YML131W | YML131W | Protein of unknown function | 2 |  | known_unknowns | c |
| 243 | YLL058W | YLL058W | Putative protein of unknown function with similarity to Str2p | 1 |  | known_unknowns | c |
| 244 | YJR061W | YJR061W | Putative protein of unknown function | 1 |  | known_unknowns | c |
| 245 | YOR364W | YOR364W | Putative protein of unknown function |  | 4 | known_unknowns | c |
| 246 | YFL015C | YFL015C | Dubious open reading frame |  | 4 | known_unknowns | c |
| 247 | YNL035C | YNL035C | Nuclear protein of unknown function |  | 4 | known_unknowns | c |
| 248 | YPR170C | YPR170C | Putative protein of unknown function |  | 3 | known_unknowns | c |
