# Supplementary figures and images for: Ribosome quality control is a central protection mechanism for yeast exposed to deoxynivalenol and trichothecin
Source: BMC Genomics. 2016 Jun 1;17:417. doi: 10.1186/s12864-016-2718-y (PMC4888481; doi:10.1186/s12864-016-2718-y)

Figure S1

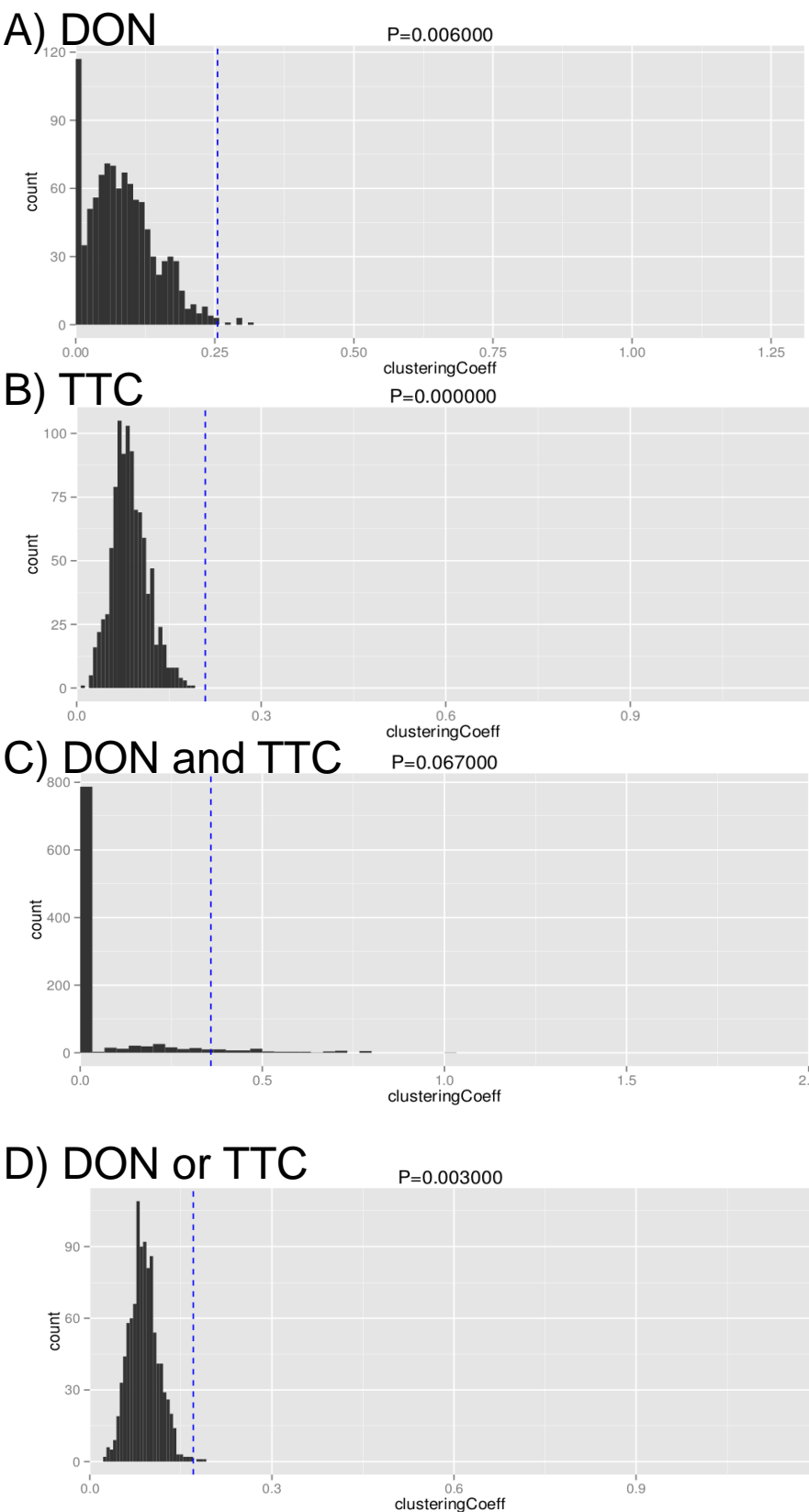

Supplement: Additional file 6: Figure S1. — The global clustering coefficient was used as a measure of the degree of clustering in the whole network. We compared the observed value (blue line) against 1000 random seed networks for A) DON, B) TTC, C) DON and Tcin, and D) DON or TTC. (PDF 588 kb) [file 12864_2016_2718_MOESM6_ESM.pdf]

Figure S2

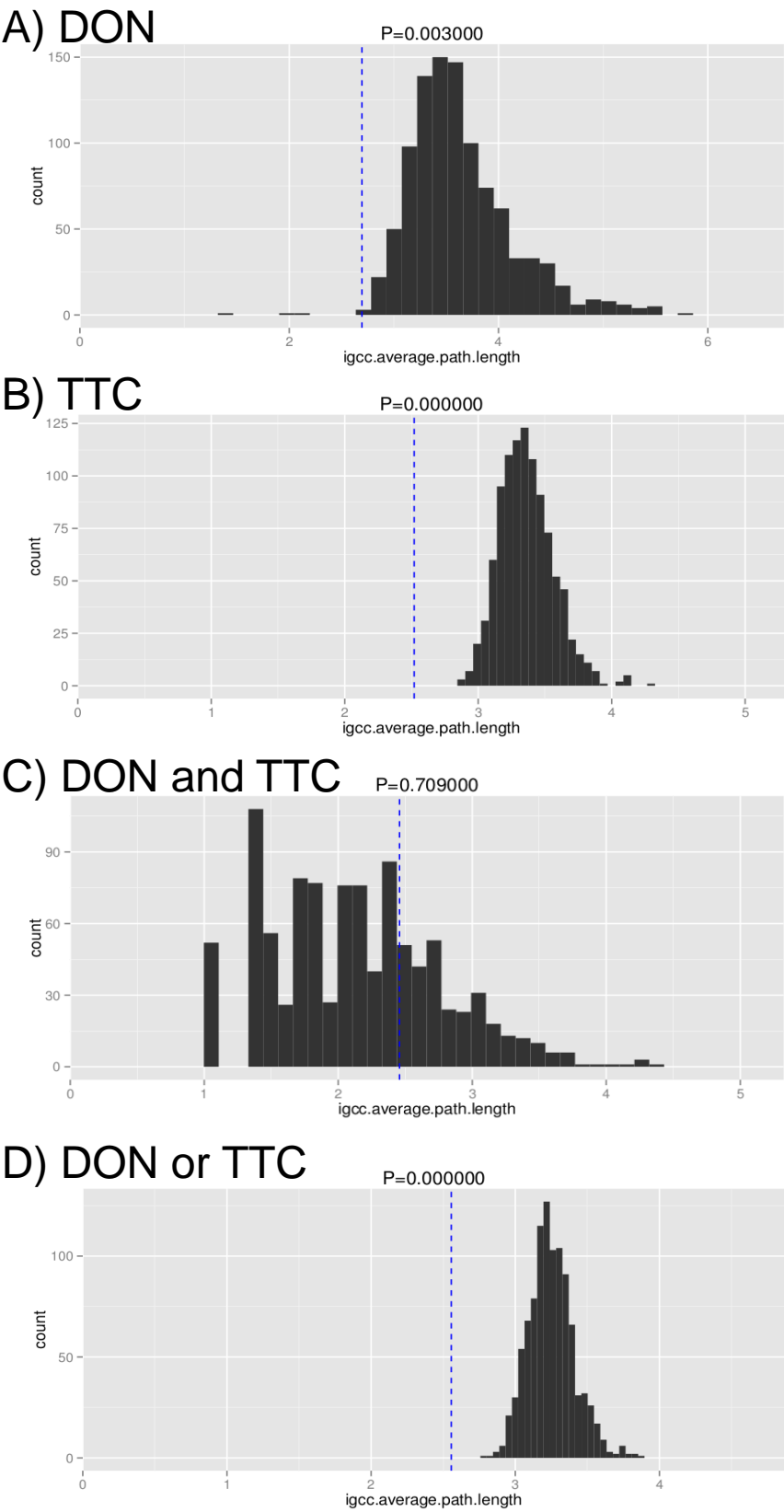

Supplement: Additional file 7: Figure S2. — The average path length of the giant connected component. We compared the observed value (blue line) against 1000 random seed networks for A) DON, B) TTC, C) DON and TTC, and D) DON or TTC. (PDF 588 kb) [file 12864_2016_2718_MOESM7_ESM.pdf]

Figure S3

A) DON

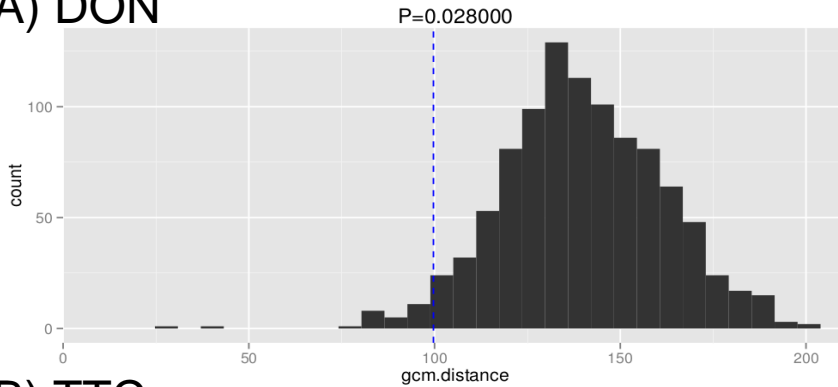

B) TTC

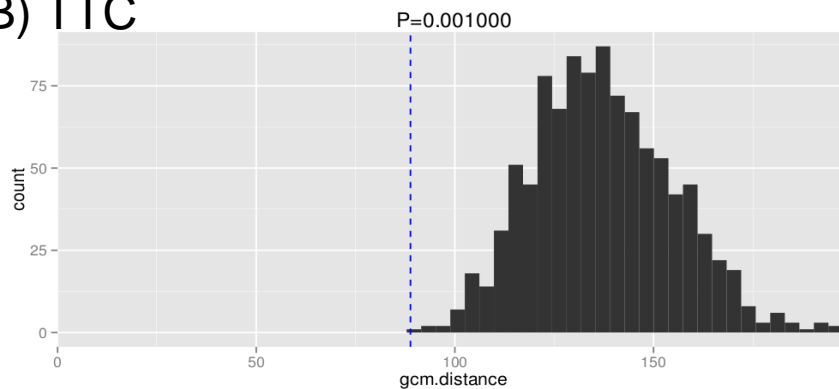

C) DON and TTC

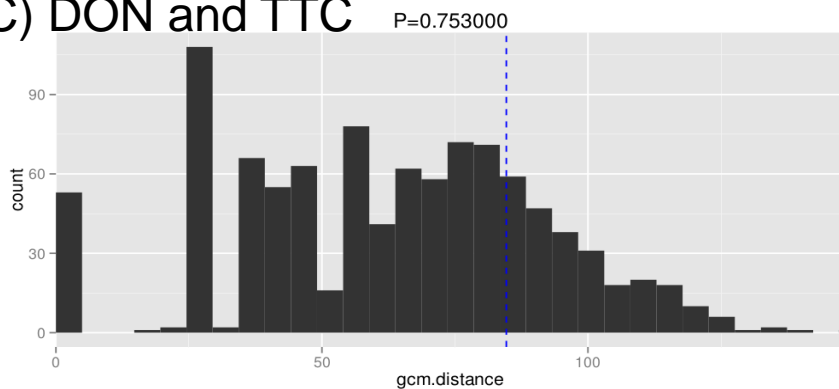

D) DON or TTC

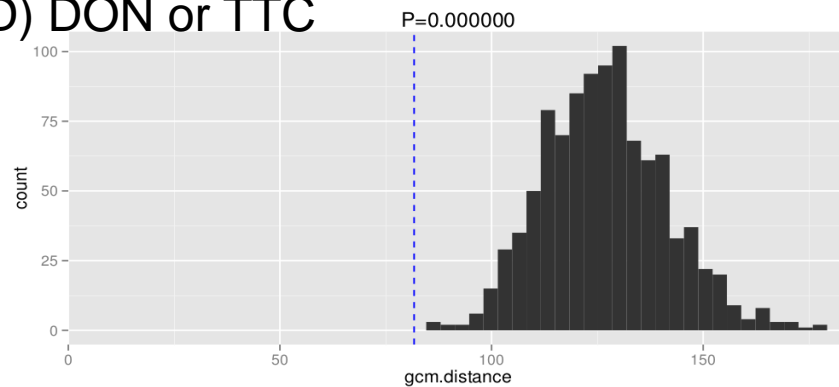

Supplement: Additional file 8: Figure S3. — Assessment of topological properties based on a Dehmer entropy measure. We compared the observed value (blue line) against 1000 random seed networks for A) DON, B) TTC, C) DON and Tcin, and D) DON or TTC. (PDF 588 kb) [file 12864_2016_2718_MOESM8_ESM.pdf]

Figure S4

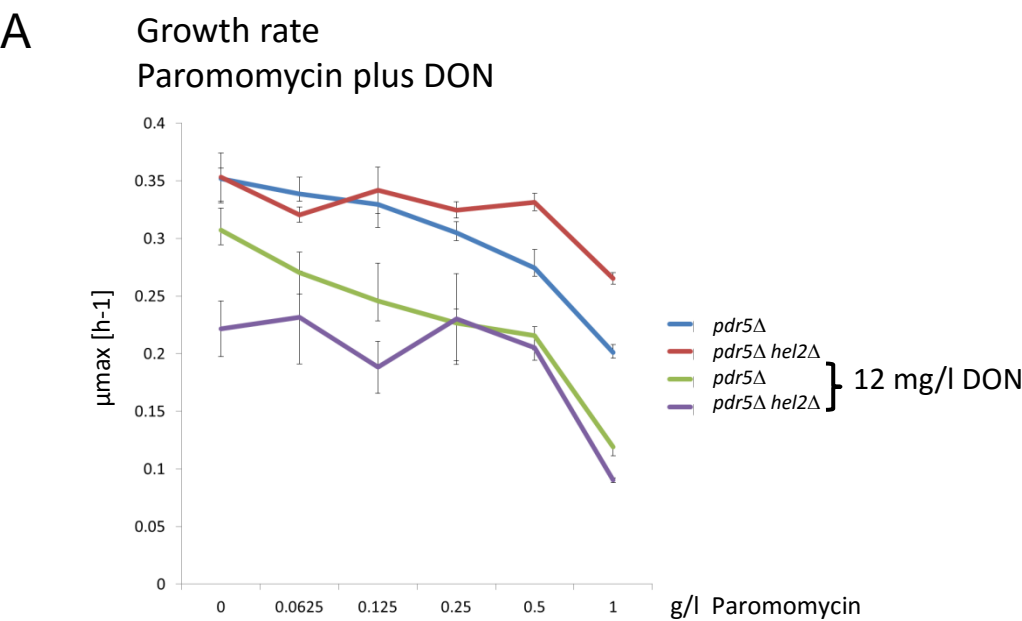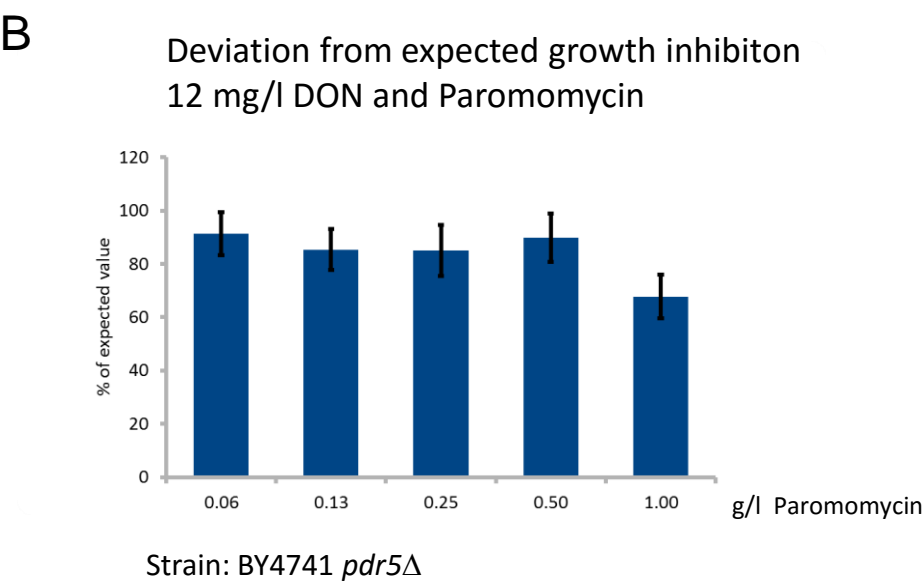

Supplement: Additional file 9: Figure S4. — Combinatorial effect of paromomycin and DON. A) Maximum growth rates determined from fitting of growth curves with grofit(R). The amount of paromomycin and the inclusion of DON are indicated. Growth was recorded in triplicates in 96 well plates in YPD in 30 min intervals. Standard deviations are indicated. B) Expected combinatorial effect of DON and paromomycin for the BY4641 pdr5Δ strain. The expected value for a multiplicative effect on growth was set to 100 %. Standard deviations are indicated. (PDF 589 kb) [file 12864_2016_2718_MOESM9_ESM.pdf]

Figure S5

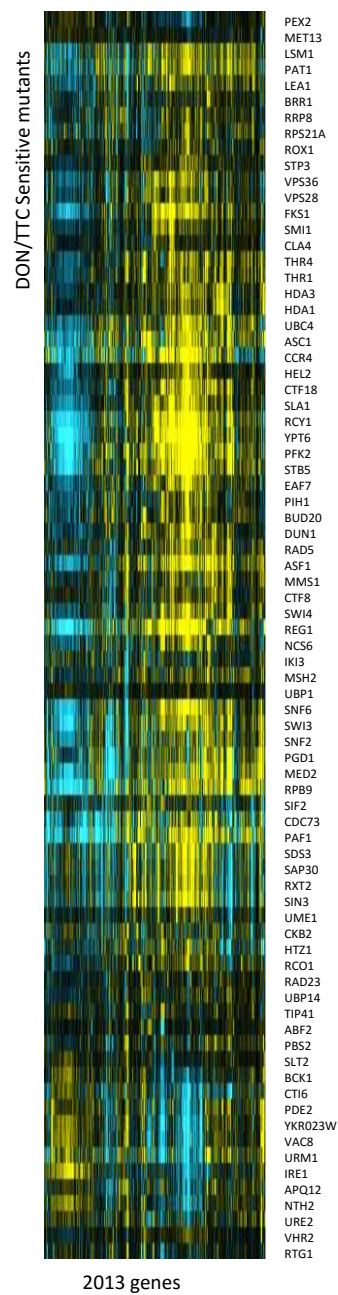

Supplement: Additional file 13: Figure S5. — Microarray data derived from O’Duibhir et al. (Molecular systems biology 2014, 10:732.) of genes identified as conferring DON and TTC resistance. Data to visualize the heat map is included in Additional file 12: Data S7. (PDF 588 kb) [file 12864_2016_2718_MOESM13_ESM.pdf]
