# Supplementary material for: Ribosome quality control is a central protection mechanism for yeast exposed to deoxynivalenol and trichothecin
Source: BMC Genomics. 2016 Jun 1;17:417. doi: 10.1186/s12864-016-2718-y (PMC4888481; doi:10.1186/s12864-016-2718-y)
Supplement: Additional file 16: Table S2. — Strains used in this study. (DOCX 17 kb) [file 12864_2016_2718_MOESM16_ESM.docx]

Supplementary Table 2 Strains used in this study

| FY1679-28C | *MAT*a *ura3-52 leu2Δ1 his3Δ200 trp1Δ63* | Delaveau et al., {Delaveau, 1994 #420} |
| --- | --- | --- |
| FY1679-28C/Δ1Δ3 | *MAT*a *ura3-52 leu2Δ1 his3Δ200 trp1Δ63Δpdr1::TRP1 Δpdr3::HIS3* | Delaveau et al., {Delaveau, 1994 #420} |
| YZGA280 | *MAT*a *ura3-52 leu2Δ1 his3Δ200 trp1Δ63 Δpdr5:hisG* | FY1679-28C, pYM31 |
| YZGA260 | *MAT*a *ura3-52 leu2Δ1 his3Δ200 trp1Δ63 Δpdr1::TRP1 Δpdr3::HIS3 (pADH-PDR5)* | FY1679-28C/Δ1Δ3  pYAK7 |
| YALA-B1 | *MAT*a *ura3-52 leu2-3,112 his3-11,115 trp1-1* | Mahe et al., {Mahe, 1996 #421} |
| YALA-G4 | *MAT*a *ura3-52 leu2-3,112 his3-11,115 trp1-1 PDR1-3* | Mahe et al., {Mahe, 1996 #421} |
| YZGA276 | *MAT*a *ura3-52 leu2-3,112 his3-11,115 trp1-1 Δpdr5::hisG* | YALA-B1, pYM31 |
| YZGA278 | *MAT*a *ura3-52 leu2-3,112 his3-11,115 trp1-1 PDR1-3 Δpdr5::hisG* | YALA-G4, pYM31 |
| Y7092 pdr5 | *MAT*alpha *can1*Δ*::STE2pr-Sp_his5 lyp1*Δ *his3*Δ*1 leu2*Δ*0 ura3*Δ*0 met15*Δ*0* | Tong et al., {Tong, 2006 #407} |
| YZGN1 | *pdr5Δ::loxP-his5^+^-loxP*, *pdr10Δ::hisG*, *pdr15*Δ::*loxP-NatMX-loxP*, *ayt1*Δ::*URA3* | This study |
| YZGN1_hel2 | *pdr5Δ::loxP-his5^+^-loxP*, *pdr10Δ::hisG*, *pdr15*Δ::*loxP-NatMX-loxP*, *ayt1*Δ::*URA3, hel2Δ::KanMX* | This study |
| RH2817 | MATα, ura3- 52, trp1::hisG | Valerius et al., 2007 {Valerius, 2007 #386} |
| RH3263 | MATα, ura3-52, trp1::hisG, leu2::hisG, asc1Δ::*LEU2* | Valerius et al., 2007 {Valerius, 2007 #386} |
| RH3510 | MATα, ura3-52, trp1::hisG, asc1-loxP SNR24 | This work |
